# Assessing early fitness consequences of exotic gene flow in the wild: a field study with Iberian pine relicts

**DOI:** 10.1111/eva.12333

**Published:** 2016-01-09

**Authors:** Gregor M. Unger, Myriam Heuertz, Giovanni G. Vendramin, Juan J. Robledo‐Arnuncio

**Affiliations:** ^1^ Department of Forest Ecology & GeneticsINIA‐CIFORMadridSpain; ^2^ INRAUMR 1202 BIOGECOCestasFrance; ^3^ University of BordeauxUMR 1202 BIOGECOTalenceFrance; ^4^ Institute of Biosciences and BioresourcesNational Research CouncilSesto Fiorentino (FI)Italy

**Keywords:** early fitness, gene flow, introgression, *Pinus pinaster*, *Pinus sylvestris*, plantations, pollen dispersal, seed dispersal

## Abstract

Gene flow from plantations of nonlocal (genetically exotic) tree provenances into natural stands of the same species is probably a widespread phenomenon, but its effects remain largely unexamined. We investigated early fitness consequences of intraspecific exotic gene flow in the wild by assessing differences in survival among native, nonlocal, and F1 intraspecific hybrid seedlings naturally established within two native pine relicts (one of *Pinus pinaster* and the other of *P. sylvestris*) surrounded by nonlocal plantations. We obtained broad‐scale temporally sequential genotypic samples of a cohort of recruits in each pine relict, from seeds before dispersal to established seedlings months after emergence, tracking temporal changes in the estimated proportion of each parental cross‐type. Results show significant proportions of exotic male gametes before seed dispersal in the two pine relicts. Subsequently to seedling establishment, the frequency of exotic male gametes became nonsignificant in *P. pinaster*, and dropped by half in *P. sylvestris*. Exotic zygotic gene flow was significantly different from zero among early recruits for *P. sylvestris*, decreasing throughout seedling establishment. Seedling mortality resulted in small late sample sizes, and temporal differences in exotic gene flow estimates were not significant, so we could not reject the null hypothesis of invariant early viability across parental cross types in the wild.

## Introduction

Global forest plantations total approximately 270·10^6^ ha, with annual planting rates of about 5·10^6^ ha in the early 21st century (FAO 2006). Planted forests provide important economic, social, and environmental services, but their potential effects on natural ecosystems also raise societal and scientific concerns (Paquette and Messier 2010). The extent and consequences of interspecific introgression between exotic tree species and their wild relatives, and of transgene escape, have drawn substantial research and political attention (e.g., reviews by Potts et al. 2003 and Sederoff 2007). Less attention has been directed toward the consequences of genetic introgression from plantations of nonlocal provenance into natural conspecific stands, although this is presumably taking place unmonitored over huge geographic areas, possibly impacting native gene pools (Potts et al. 2003; Lefèvre 2004; Laikre et al. 2010). The term ‘intraspecific exotic gene flow’ or ‘intraspecific exotic introgression’ is used here to refer to gene movement from nonlocal (genetically exotic) populations of a species into nearby natural populations of the same species, following Laikre et al. (2010). The latter point out that genetic monitoring of this process within forestry research and management is urgent but remains virtually nonexistent, in contrast with decades‐long studies in fisheries. As far as we know, we are the first to address, in forest trees, the empirical consequences of intraspecific gene flow from exotic plantations in the wild.

Intraspecific exotic gene flow monitoring requires both (i) quantifying the exposure of native forests to effective pollen and seed immigration from exotic stands, and (ii) assessing the consequences of exotic gene flow for native gene pools. Notwithstanding interspecific, demographic, and environmental variation, it is becoming clear from empirical and modeling studies that the high fecundity and efficient dispersal syndromes of trees will generally allow non‐negligible exposure of native stands to pollen (and possibly seed) dispersal from conspecific plantations, unless isolation distances are in the order of tens or even hundreds of kilometers (Smouse et al. 2007; Robledo‐Arnuncio et al. 2010). Genetic and phenological barriers to conspecific mating between native and exotic trees growing in the same environment are unlikely to be strong (González‐Martínez et al. 2005), and the frequency of effective exotic pollination will thus largely depend on geographical isolation and the relative number of pollen donors in exotic and native stands (Ellstrand and Elam 1993; Kremer et al. 2012). Well‐established methods are available to quantify the frequency of contemporary effective dispersal among populations. In particular, genetic assignment methods allow tracing the ancestry of individuals to more than one population (Pritchard et al. 2000; Anderson and Thompson 2002) or explicitly estimate recent gene flow rates among population pairs (Wilson and Rannala 2003; Faubet and Gaggiotti 2008; Broquet et al. 2009), with the possibility to dissect the latter into seed‐ and pollen‐mediated components (Robledo‐Arnuncio 2012; Unger et al. 2014). Recent empirical studies using these methods have proved their practical utility, revealing substantial introgression rates from exotic plantations into native conspecific stands of several temperate tree species, mainly through pollen flow (Robledo‐Arnuncio et al. 2009; Millar et al. 2012; Steinitz et al. 2012; Unger et al. 2014).

The exposure of native gene pools to exotic gene flow from conspecific plantations seems generally unavoidable, but the adaptive consequences are not as clear. One of the reasons for a negative social perception of exotic plantations is their presumed harmful effect on native genetic resources. Seed dispersal from plantations may result in the establishment of exotic individuals in the wild, which may gradually displace local trees if propagule pressure is sufficiently large, irrespective of potential fitness differences between local and exotic genotypes in the local environment (Lenormand 2002). In addition, intraspecific hybrids from mating between native and exotic individuals may exhibit reduced fitness (outbreeding depression) if they lose local alleles with positive additive effects on fitness and/or if recombination disrupts positive epistatic interactions among local alleles at different loci (Keller et al. 2000; Tallmon et al. 2004). However, intraspecific hybrids can also experience enhanced fitness (heterosis), via increased heterozygosity and the masking of deleterious recessive alleles (Tallmon et al. 2004; Whiteley et al. 2015). The genetic mechanisms underlying the opposing forces of outbreeding depression and heterosis may operate simultaneously, and whether positive or negative effects prevail will depend, among other factors, on the levels of individual inbreeding and among‐population genetic divergence, the genetic architecture of local adaptation, the number of hybrid generations, and the environmental conditions. For instance, heterosis tends to be stronger for inbred recipient plant populations (Willi et al. 2007; Pickup et al. 2013) and for F1 hybrids, which experience the greatest increase in heterozygosity (Tallmon et al. 2004). On the other hand, increasing outbreeding depression is expected for greater genetic divergence between parental populations (Willi and Van Buskirk 2005), stronger positive epistasis among local alleles, and increasing recombination in the F2 and subsequent hybrid generations (Fenster and Galloway 2000), especially when fitness is measured in the wild (Whiteley et al. 2015).

Over generations, the effective immigration of genes from genetically exotic plantations may reduce local adaptation of natural populations by causing deviation of phenotypes from their local optimum, via allelic frequency changes opposite to natural selection and/or breakdown of co‐adapted gene complexes (Lenormand 2002; Savolainen et al. 2007). Negative adaptive effects are expected to increase for high levels of gene flow and strong adaptive divergence between populations (García‐Ramos and Kirkpatrick 1997; Garant et al. 2007; Lopez et al. 2008). But exotic gene immigration may also help natural populations adapt to their native environment, by alleviating inbreeding depression and augmenting the genetic variance available for selection (Gomulkiewicz et al. 1999; Ingvarsson 2001; Whiteley et al. 2015), especially for low levels of gene flow, small recipient populations, and temporally changing environments (Alleaume‐Benharira et al. 2006; Duputié et al. 2012). The longer‐term empirical evidence available comes from salmonid studies, which showed that introgressed genes from exotic conspecific populations were selected against in the wild (Hansen et al. 2010), and can result in loss and altered transcription patterns of locally adaptive genetic variation (Roberge et al. 2008; Bourret et al. 2011) and ultimately in reduced survival, recruitment, and adaptability to changing conditions of wild populations (McGinnity et al. 2009).

Initial steps in genetic monitoring of exotic gene flow should assess the relative fitness of native individuals versus pure exotic immigrants, F1 native‐exotic hybrids, and later generation recombinants. This is obviously a daunting task in long‐living species such as trees, where lifetime fitness measurements are unfeasible in general even within a single generation. However, estimates of major fitness components also give valuable insights (Lande and Arnold 1983), and easily measurable early components such as seedling viability not only make a major contribution to tree fitness (Petit and Hampe 2006), but also to tree range limits (Jackson et al. 2009). In addition, several experimental approaches should prove useful for informing management strategies within a reasonable time frame. A first standard option is to obtain controlled‐pollinated seeds of all feasible cross types (typically only parental and F1) and grow them in common laboratory or garden environments (e.g., Ramírez‐Valiente and Robledo‐Arnuncio 2014). The advantages of this option are that it allows replicated balanced designs with unambiguous knowledge of individual parental origin and age, minimizes the confounding effect of background environmental variation on estimates of fitness differences among cross types (Rausher 1992), and permits investigating differential responses of progenies to specific environmental factors of interest. However, this option is costly, and the important germination and initial establishment phases are typically conducted in unrealistically optimal conditions, so as not to compromise necessary sample sizes. Moreover, the laboratory or garden environments may poorly reflect the ensemble of interacting biotic and abiotic factors present in the wild, potentially resulting in artificial measures of both outbreeding and inbreeding depression (Whiteley et al. 2015).

The disadvantages of laboratory and common garden experiments are largely circumvented by a second complementary approach: Assuming it is feasible to ascertain the contribution of native versus exotic parents to a sample of individuals without pedigree data (e.g., using the genetic methods mentioned above), it would then be possible to measure fitness differences across the parental cross types identified among standing wild individuals (much as in admixed‐stock fish studies, e.g., Le Cam et al. 2015). Sampled individual trees should be selected and categorized by age, to account for fitness trait variation across ontogenetic stages (Lande and Arnold 1983), and sampled from the same area in sufficient numbers to reduce differences in average micro‐environmental conditions experienced by different parental cross types. This approach may enable immediate field measurements of hybrid fitness for F2 and later recombinants in cases where exotic tree plantations were introduced sufficiently long ago. Most forest plantations are relatively recent, however, and therefore large age differences between parental cross types will generally restrict meaningful comparisons to early stage fitness traits, for example, if introgression is incipient and only young F1 hybrid recruits are present along with pure adult parents and nonhybrid offspring.

Within this framework, we investigated two separate cases, one in *Pinus pinaster* Ait. and the other in *P. sylvestris* var. *nevadensis* D.H. Christ, of incipient genetic introgression from nonlocal tree plantations into Iberian native relict populations of the same species inhabiting marginal xeric environments. We employed an original field approach, in which we assessed differences in early survival (a major early component of fitness) among native, exotic, and F1 hybrid seedlings naturally established under the canopy of the native stands. In particular, we obtained temporally sequential samples of a cohort of recruits throughout a contemporary recruitment season, tracking changes in the relative proportion of each parental cross types from seeds before dispersal to established seedlings months after emergence, a period during which strong selective filters operate for tree species (Petit and Hampe 2006), especially in Mediterranean environments (Castro et al. 2004). In this way, we tested the null hypothesis of invariant early survival rates between native and exotic seedlings, as well as between each pure parental class and hybrids. If native individuals were locally adapted, we would expect higher survival rates for native than for pure exotic seedlings. Higher survival for native than for hybrid seedlings would further suggest that local adaptation results in outbreeding depression (i.e., that exotic gene flow deviates phenotypes from what is optimal for the local xeric environments) and that inbreeding depression is either absent or comparatively weak. By contrast, if native individuals were suffering from significant inbreeding depression, a possibility given the small size of the two native relicts, we might then observe significantly higher survival rates for hybrid (and even for pure exotic) seedlings than for native seedlings.

## Material and methods

### Study sites and sampling

**Figure 1:**
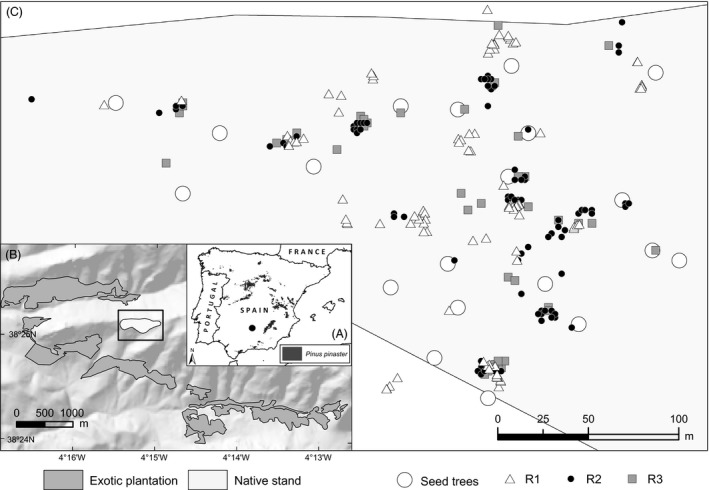
Maps of *Pinus pinaster* study site in Fuencaliente (Spain) showing: (A) the location of Fuencaliente (marked with a dot) and the distribution of native populations of the species within the Iberian Peninsula, (B) a digital elevation model of the study area and the distribution of exotic plantations (in dark grey) and the native stand (in light grey, and framed), and (C) the distribution of sampled seed–trees and sequential recruit samples (R1, R2, and R3) within the boundaries of the native stand (in light gray).

**Figure 2:**
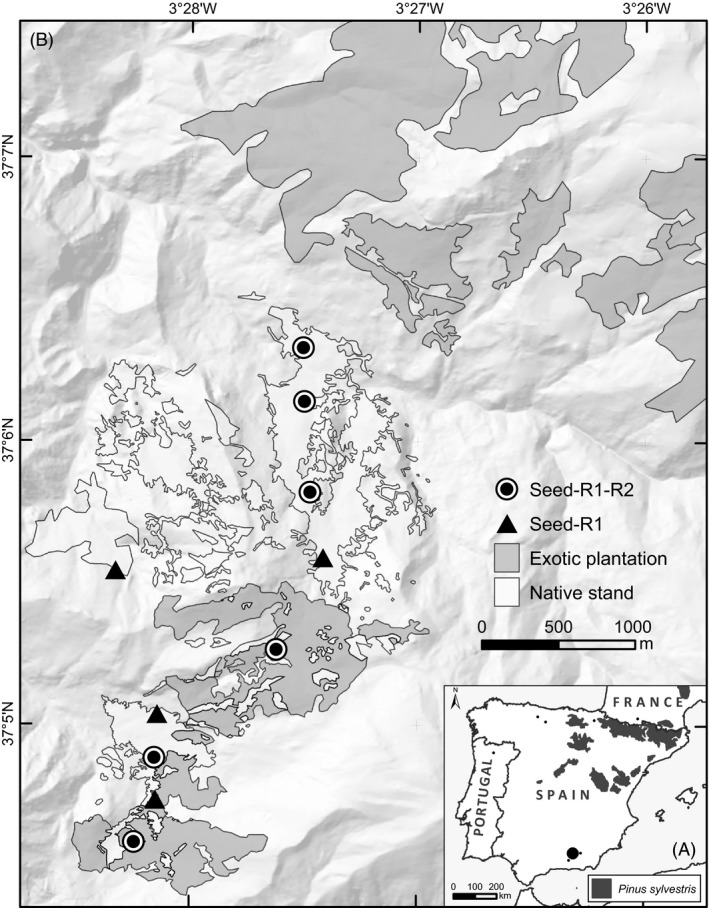
Maps of *Pinus sylvestris* study site in Sierra Nevada National Park (Spain) showing: (A) the location of Sierra Nevada (marked with a dot) and the distribution of native populations of the species within the Iberian Peninsula, and (B) a digital elevation model of the study area and the distribution of exotic plantations (in dark grey) and native stands (in light grey). Sampling plots within the native stand are indicated by circles (seed and two sequential seedling samples available) and triangles (seed and one sequential seedling sample available).

We studied two Iberian relict tree populations, one of *Pinus pinaster* at Fuencaliente (38°25′N, 4°15′W, Fig. 1) and the other of *P. sylvestris* var. *nevadensis* at Sierra Nevada National Park (37°5′N, 3°28′W, Fig. 2). As described in more detail in Unger et al. (2014), the two small relicts are strongly isolated from other native conspecific populations (over 100 km away for *P. pinaster*, 50–350 km away for *P. sylvestris*), but they are closely surrounded by widespread conspecific exotic plantations established about 50–60 years ago. Similarly for both species, up to 6–8% of established saplings that have dispersed from local mothers during the last 15–30 years have been sired by exotic fathers, whereas no significant seed immigration from the plantations has been found over the same time period (Unger et al. 2014). Interestingly, the proportion of exotic pollen flow at the seed stage (before dispersal) during a single more recent dispersal season was significantly higher for *P. sylvestris*, as much as 40% (Robledo‐Arnuncio et al. 2009). This difference could be due to (i) a postdispersal selective disadvantage of intraspecific hybrids, and/or (ii) stochastic interannual pollen flow variation, or (iii) a progressive increase in the relative amount of exotic pollen received by native mothers as plantations reached full reproductive competence. We test here the hypothesis of selective differences between native and intraspecific hybrid genotypes in the wild.

Both species are monoecious, predominantly outcrossing, wind‐pollinated, and anemochorous. In the Iberian Peninsula, seed dispersal spans from May to October for *P. pinaster* (Juez et al. 2014) and from January to March for *P. sylvestris* (Castro et al. 1999). Seedling emergence subsequently starts with the first rains after the summer drought (October–November) for *P. pinaster* and with late‐spring warmth (May) for *P. sylvestris* (personal observations; Castro et al. 1999). After emergence, early seedling mortality is severe for both species, over 90% during the first year (Castro et al. 2004; Vizcaíno‐Palomar et al. 2014). We collected sequential offspring samples spanning the first months of a contemporary recruitment season, before and after seed dispersal, for both species. Specifically, we collected mature open‐pollinated cones before seed dispersal from 20 native seed‐trees of each species (‘seed’ samples), in April 2010 for *P. pinaster* and in December 2010 for *P. sylvestris*. Sampled seed‐trees were located over the whole area of the relict stands (0.12 km² in *P. pinaster*, 1.39 km² in *P. sylvestris*; Figs 1 and 2), distributed in ten pairs in the case of *P. sylvestris* (Fig. 2). Immediately after the ensuing seed dispersal period, we sampled *n*_R1_ naturally established, recently emerged seedlings (‘R1’ samples; *n*_R1_ = 101 seedlings in November 2010 for *P. pinaster*, and *n*_R1_ = 217 seedlings in late May 2011 for *P. sylvestris*), which were collected where found over the entire relict stand for *P. pinaster* (Fig. 1) and within 50 m of sampled seed‐tree pairs for *P. sylvestris* (Fig. 2). Subsequent (‘R2’) samples of the same recruitment cohort of each species were taken in late winter for *P. pinaster* (*n*_R2_ = 109, March 2011), and in the peak of summer for *P. sylvestris* (*n*_R2_ = 41, August 2011). The R2 samples represented an exhaustive collection of all seedlings found across the same areas where R1 seedlings were sampled (Figs 1 and 2). The smaller size and incomplete spatial coverage of *P. sylvestris*'s R2 sample resulted from severe mortality during a very dry and hot summer; no surviving seedlings were found for this species after the summer in the study area. We were able, however, to collect an additional (‘R3’) seedling sample for *P. pinaster* in mid‐summer (only *n*_R3_ = 45 remaining seedlings were found over the entire area in July 2011; Fig. 1).

Seedling samples were placed in paper envelopes, dried on silica gel, and stored at room temperature in the laboratory until DNA extraction. Collected cones were stored at 4°C until opening was induced by placing them at room temperature (a few cones remained closed and were oven dried for 12–20 h at 35–40°C), after which seeds were extracted manually, cleaned, and kept in paper envelopes at 4°C till inducing germination. For each species, 20–120 randomly sampled seeds from each of 20 seed‐trees were surface‐sterilized in 0.35% sodium hypochlorite for 5 min and washed with distilled water. Seeds were then placed on quantitative filter paper humidified with fungicide (3 g/L of Captan) within sealed Petri dishes, and incubated at 22°C and 24 h of light to induce germination. Germinated seedlings were transferred to trays with perlite and kept in the germination chambers under the same conditions until DNA extraction. Final seed sample sizes for analysis were *n*_*S*_ = 657 for *P. pinaster* and *n*_*S*_ = 400 for *P. sylvestris*.

### Laboratory analysis

We extracted DNA from all 1570 offspring samples (912 for *P. pinaster* and 658 for *P. sylvestris*) using the Invisorb DNA Plant HTS 96 Kit (STRATEC Molecular GmbH, Berlin, Germany) and 20 mg of dried or 30 mg of fresh needle tissue. The samples were screened with a combination of paternally inherited chloroplast microsatellites (cpSSRs) and biparentally inherited nuclear microsatellites (nSSRs). In particular, we genotyped *P. pinaster* samples at six cpSSRs and twelve nSSRs, and *P. sylvestris* samples at seven cpSSRs and ten nSSRs. Used markers were the same as those successfully amplified and scored by Unger et al. (2014), who present a detailed list of loci along with protocols for amplification and fragment size analysis.

To estimate exotic introgression rates among sampled offspring, we also employed the reference native and exotic adult genotypic samples obtained by Unger et al. (2014) from the same study areas using the same loci (*n*_*N*_ = 101 native trees and *n*_*P*_ = 139 exotic trees for *P. pinaster*;* n*_*N*_ = 202 and *n*_*P*_ = 193 for *P. sylvestris*).

### Analysis of genetic diversity and differentiation

For nSSR loci, we computed observed (*H*_*o*_) and expected (*H*_*e*_; Nei 1987) heterozygosities and inbreeding coefficients (*F*_IS_), and performed locus‐by‐locus exact tests of Hardy–Weinberg proportions (via the Markov‐chain algorithm of Guo and Thompson 1992; run for 10^7^ steps after 10^6^ dememorization steps), using arlequin ver. 3.5.1.2 software (Excoffier and Lischer 2010). Additionally, we calculated observed and effective (Nielsen et al. 2003; eqn 16) numbers of cpSSR haplotypes and nSSR alleles. The population inbreeding model in INest ver. 1.0 software (Chybicki and Burczyk 2009) was used to jointly estimate null allele frequencies and inbreeding coefficients for nSSR loci, which provides information about the relative effect of null alleles versus actual inbreeding on observed deviations from Hardy–Weinberg proportions. Genetic differentiation between samples was computed using both the amova‐based *F*_ST_‐statistic implemented in arlequin software and Jost's bias‐corrected differentiation index (*D*_est_; Jost 2008) implemented in demetics (Gerlach et al. 2010) and spade (Chao and Shen 2010). We tested whether differentiation estimates were significantly different from zero by randomly permuting genotypes among samples 10 000 times.

### Estimation of introgression rates

We estimated contemporary gene flow rates from the exotic plantations into the native relicts using the maximum‐likelihood model introduced by Unger et al. (2014). The approach uses genetic information to ascertain population membership of the parents of a sample of contemporary offspring and, analogous to other assignment methods (Manel et al. 2005), does not require migration–drift equilibrium assumptions. In particular, the model makes use of a combination of uni‐ and biparentally inherited markers to dissect contemporary gene flow rates into zygotic, male gametic, and female gametic components. Each seedling sampled under the native stand canopy (see below the special treatment for seeds collected before dispersal) can belong to one of four groups: seedlings from native mothers and exotic fathers, with proportion *m*_*m*_ (the male gametic gene flow rate); seedlings from two exotic parents, with proportion *m*_*z*_ (the zygotic gene flow rate); seedlings from exotic mothers and native fathers, with proportion *m*_*f*_ (the female gametic gene flow rate); or seedlings from two native parents, with proportion (1 − *m*_*m*_ − *m*_*z*_ − *m*_*f*_). Given the vector of observed seedling chloroplast–nuclear multilocus genotypes **G**_**R**_, and the reference samples of *n*_*P*_ exotic and *n*_*N*_ native adult genotypes, *m*_*m*_, *m*_*z*_, and *m*_*f*_ are estimated by maximizing the likelihood:

(1) $$\begin{array}{cc} L(m_{m},m_{z},m_{f}| & n_{P},n_{N},G_{R})\\ & =\prod_{G_{R}=1}^{K_{R}}\mathrm{Pr}{\left(G_{R}|m_{m},m_{z},m_{f},n_{P},n_{N}\right)}^{n_{GR}} \end{array}$$

where *K*_*R*_ is the observed number of different combined chloroplast–nuclear genotypes in the seedling sample and *n*_*GR*_ is the number of seedlings carrying the *G*_*R*_‐th genotype. The probability of observing seedling genotype *G*_*R*_ is estimated as

(2) $$\begin{array}{cc} \mathrm{Pr}(G_{R}|m_{m},m_{z},m_{f},n_{P},n_{N})=\; & m_{m}{\text{Pr}}_{PN}\left(G_{R}|n_{P},n_{N}\right)\\ & +m_{z}{\text{Pr}}_{PP}\left(G_{R}|n_{P}\right)\\ & +m_{f}{\text{Pr}}_{NP}\left(G_{R}|n_{P},n_{N}\right)\\ & +[1-\left(m_{m}+m_{z}+m_{f}\right)]\\ & \;{\text{Pr}}_{NN}\left(G_{R}|n_{N}\right) \end{array}$$

where Pr_*PN*_(*G*_*R*_¦*n*_*P*_, *n*_*N*_), Pr_*PP*_(*G*_*R*_¦*n*_*P*_), Pr_*NP*_(*G*_*R*_¦*n*_*P*_, *n*_*N*_) and Pr_*NN*_(*G*_*R*_¦*n*_*N*_) are the probabilities that a seedling has genotype *G*_*R*_ if it was born to a native mother pollinated by an exotic father, two exotic parents, an exotic mother pollinated by a native father, or two native parents, respectively, given the reference adult genotypic frequencies estimated from the *n*_*P*_ exotic and *n*_*N*_ native adults. These combined chloroplast–nuclear multilocus genotypic probabilities are calculated as the product of independent haplotypic probabilities for cpSSR haplotypes and single‐locus genotypic probabilities for nSSR loci, assuming that nuclear loci are unlinked and in Hardy–Weinberg equilibrium (see Supporting Information Annex S1 for details). All the nSSR loci used to estimate gene flow rates met the Hardy–Weinberg equilibrium assumption (see Appendix S2 in Unger et al. 2014). The employed maximum‐likelihood method and molecular markers are expected to yield generally minimally biased estimates of exotic gene flow components, as shown by a numerical simulation study (Unger et al. 2014). Additional cross‐validation tests conducted here are consistent with these predictions (see Supporting Information Annex S2).

In the special case of seed samples collected from native mothers before dispersal, individuals can only belong to one of two categories: seeds from native mothers and exotic fathers (with proportion *m*_*m*_) or seeds from two native parents (with proportion 1−*m*_*m*_). Given the vector of observed chloroplast–nuclear multilocus seed genotypes **G**_**S**_, eqns (1) and (2) simplify then to

(3) $$L\left(m_{m}|n_{P},n_{N},G_{S}\right)=\prod_{G_{S}=1}^{K_{S}}{\mathrm{Pr}(G_{S}|m_{m},n_{P},n_{N})}^{n_{GS}}$$

and

(4) $$\begin{array}{cc} \mathrm{Pr}(G_{S}|m_{m},n_{P},n_{N}) & =m_{m}{\text{Pr}}_{PN}\left(G_{S}|n_{P},n_{N}\right)\\ & \;+\left(1-m_{m}\right){\text{Pr}}_{NN}\left(G_{S}|n_{N}\right) \end{array}$$

from which it is possible to obtain estimates of *m*_*m*_ for seeds before dispersal. We used eqns (1) and (2) to estimate *m*_*m*_, *m*_*z,*_ and *m*_*f*_ for every sequential seedling sample of each species, and eqns (3) and (4) to estimate *m*_*m*_ for each of the two seed samples, obtaining confidence intervals using the profile‐likelihood method. Note that the seedling‐stage *m*_*m*_ in eqn (1) is post‐seed dispersal (i.e., a proportion relative to the total sample of seedlings, dispersed from both local and exotic mothers), while the seed‐stage *m*_*m*_ in eqn (3) is pre‐seed dispersal (i.e., relative to offspring from local mothers only). In order to allow meaningful comparison between the two, we divided the seedling‐stage parameter *m*_*m*_ by (1 − *m*_*z*_ − *m*_*f*_), a transformation that yields the proportion of male gametic immigrants among established seedlings relative to the fraction of seedlings dispersed from local mothers only. Hereafter, seedling‐stage *m*_*m*_ will always refer to the latter transformation.

### Testing differences in exotic introgression over sequential offspring samples

We tested the null hypothesis that the proportion of exotic immigrants within the contemporary offspring cohort under study remains constant in time. In particular, we tested for differences in exotic gene flow rates *m*_*m*_, *m*_*z,*_ and/or *m*_*f*_ between each pair of seedling versus seedling samples for each species (e.g., R1 vs R2, R1 vs R3, and R2 vs R3 in *P. pinaster*), and additionally for differences in *m*_*m*_ between each pair of seed versus seedling samples (e.g., seeds vs R1 and seeds vs R2 in *P. sylvestris*). To compare seedling versus seedling samples, we used randomization tests in which observed differences in exotic gene flow rates between two samples of size *n*_1_ and *n*_2_ are compared with the distribution of differences obtained (under the null hypothesis) by applying eqn (1) to 1000 random allocations of the *n*_1_+*n*_2_ seedlings to two samples of size *n*_1_ and *n*_2_. This assumption‐free nonparametric test (Manly 1997) is not justified; however, for the case of seeds versus seedling sample comparisons because, even under the relevant null hypothesis of equal *m*_*m*_, the elements in the two samples are dissimilar (i.e., from different parental origins, unless seedlings satisfied *m*_*z*_ = *m*_*f*_ = 0) and need thus to be analyzed with different models (eqn (1) vs eqn (3)). A possible alternative is a bootstrap‐based two‐sample comparison, but bootstrapping can seriously underestimate the variance of gene flow rate estimates, especially for small samples (Robledo‐Arnuncio et al. 2009; J.J. Robledo‐Arnuncio and G.M. Unger, unpublished data). Therefore, we tested for differences in *m*_*m*_ between seed and seedling samples using likelihood‐ratio statistics (see Clark et al. 1998). Assuming that a given pair of seed‐seedling samples has equal *m*_*m*_, we obtained parameter estimates based on the joint likelihood of the two samples:

(5) $$\begin{array}{cc} L_{A} & =L(m_{m},m_{z},m_{f}|n_{P},n_{N},G_{R},G_{S})\\ & =\prod_{G_{R}=1}^{K_{R}}{\mathrm{Pr}(G_{R}|m_{m},m_{z},m_{f},n_{P},n_{N})}^{n_{GR}}\\ & \;\prod_{G_{S}=1}^{K_{S}}{\mathrm{Pr}(G_{S}|m_{m},n_{P},n_{N})}^{n_{GS}} \end{array}$$

We then assumed that *m*_*m*_ varies between the seedling (*m*_*m*,*R*_) and seed (*m*_*m*,*S*_) samples, and obtained parameter estimates based on the likelihood

(6) $$\begin{array}{cc} L_{B} & =L(m_{m,R},m_{z},m_{f},m_{m,S}|n_{P},n_{N},G_{R},G_{S})\\ & =\prod_{G_{R}=1}^{K_{R}}{\mathrm{Pr}(G_{R}|m_{m,R},m_{z},m_{f},n_{P},n_{N})}^{n_{GR}}\\ & \;\prod_{G_{S}=1}^{K_{S}}{\mathrm{Pr}(G_{S}|m_{m,S},n_{P},n_{N})}^{n_{GS}} \end{array}$$

The deviance *D *=* *−2 ln (*L*_*A*_/*L*_*B*_) is asymptotically distributed as *χ* ^2^ with one degree of freedom. Large deviances mean that the inequality *m*_*m*,*R*_ ≠ *m*_*m*,*S*_ improves the likelihood of the data so substantially that the null hypothesis of equal male gametic introgression can be rejected. We corrected for multiple testing using the Holm–Bonferroni correction (Holm 1979). All calculations were conducted using C++ scripts available from JJRA.

## Results

### Population differentiation and genetic diversity

Genetic divergence between native and exotic adult trees has previously been shown to be marked and significant both for nSSR and cpSSR loci (*F*_ST_ = 0.083–0.112 and *D*_est_ = 0.154–0.794 for *Pinus pinaster*,* F*_ST_ = 0.044–0.046 and *D*_est_ = 0.089–0.726 for *P. sylvestris*; results from Unger et al. 2014 are included for reference in Table 1). We found here similar levels of divergence between exotic adults and each of the sequential offspring samples collected within the two native stands (Table 1), although with somewhat higher values in the case of *P. pinaster* (*F*_ST_ = 0.102–0.181 and *D*_est_ = 0.170–0.848) and slightly lower ones for *P. sylvestris* (*F*_ST_ = 0.016–0.039 and *D*_est_ = 0.055–0.646). The level of genetic divergence between native adult trees and the sequential offspring samples was either nonsignificant or low when significant, a result similar across species, marker types, and measures of differentiation (Table 1). Exotic adults had a high cumulative frequency of private cp‐haplotypes that were not detected among native adults (61% in *P. pinaster* and 88% in *P. sylvestris*; Supporting Information Tables S1 and S2). These plantation‐specific cp‐haplotypes were also detected among sequential offspring samples at cumulative frequencies that were low (0–4%) in *P. pinaster* and somewhat higher (7–13%) in *P. sylvestris* (Supporting Information Tables S1 and S2). Across nSSR loci, the average cumulative frequency of plantation‐specific alleles was 5.2% in *P. pinaster* and 5.8% in *P. sylvestris*, and these alleles were also present at low cumulative frequencies among sequential offspring samples (0.2–0.8% in *P. pinaster* and 0.5–1.0% in *P. sylvestris*; Supporting Information Tables S3 and S4).

**Table 1:** Genetic differentiation estimates between different sample pairs for *Pinus pinaster* at Fuencaliente and for *P. sylvestris* at Sierra Nevada National Park

Species		cpSSRs		nSSRs	
	Sample pair	*F*_ST_	*D*_est_	*F*_ST_	*D*_est_
*P. pinaster*	NA – EA	0.112	0.794	0.083	0.154
	NA – S	−*0.002*	−*0.006*	0.004	0.006
	NA – R1	−*0.002*	−*0.006*	0.013	0.019
	NA – R2	*0.006*	*0.013*	0.011	0.015
	NA – R3	*0.019*	*0.043*	0.012	0.015
	EA – S	0.125	0.814	0.102	0.170
	EA – R1	0.136	0.823	0.109	0.188
	EA – R2	0.172	0.835	0.105	0.172
	EA – R3	0.181	0.848	0.110	0.184
*P. sylvestris*	NA – EA	0.044	0.726	0.046	0.089
	NA – S	0.006	*0.070*	0.006	0.009
	NA – R1	0.009	0.113	0.003	0.003
	NA – R2	*0.010*	*0.096*	*0.007*	0.011
	EA – S	0.023	0.646	0.039	0.082
	EA – R1	0.016	0.586	0.038	0.080
	EA – R2	0.020	0.566	0.032	0.055

NA, EA, native and exotic adults, respectively; S, seed sample before dispersal; R1, R2, R3, temporally sequential recruit (seedling) samples; *F*_ST_, AMOVA‐based population differentiation index; *D*_est_, Jost's bias‐corrected differentiation index. Estimates in italics are not significantly different from zero (*α *= 0.05).

The genetic diversity of exotic plantations has previously been found to be higher than that of native adult trees for both species, more markedly in terms of effective numbers of cpSSR haplotypes than in terms of nSSR heterozygosities or effective numbers of alleles (results from Unger et al. 2014 are displayed in Table 2 for reference). Sequential offspring samples collected from native stands in the present study showed either lower genetic diversity estimates than both native and exotic adults (in *P. pinaster*) or intermediate values between the two (in *P. sylvestris*) (Table 2). Average *F*_IS_ values across nSSR loci were positive for the two species in all samples, with larger estimates for offspring than for adults, but all estimates dropped to low nonsignificant values when accounting for the presence of null alleles, suggesting no actual inbreeding (Table 2).

**Table 2:** Genetic diversity estimates for *Pinus pinaster* at Fuencaliente and for *P. sylvestris* at Sierra Nevada National Park

Species	Sample	*n*	*nh*	*nh*_*e*_	*na*	*na*_*e*_	*H*_*o*_	*H*_*e*_	*F*_IS_	*F*’_IS_	*f*(null)
*P. pinaster*	Native adults	101	15	4.01	5.08	2.56	0.507	0.548	0.075 (3)	0.000	0.030
	Exotic adults	139	59	32.66	7.58	3.39	0.586	0.646	0.093 (2)	0.000	0.043
	Seeds	657	40	4.07	6.33	2.38	0.485	0.532	0.088 (6)	0.008	0.028
	Recruits‐R1	101	15	3.36	4.92	2.27	0.449	0.507	0.114 (1)	0.022	0.025
	Recruits‐R2	109	9	2.65	4.25	2.20	0.417	0.480	0.132 (1)	0.000	0.045
	Recruits‐R3	45	8	2.33	4.17	2.19	0.415	0.459	0.098 (0)	0.000	0.036
*P. sylvestris*	Native adults	202	23	9.07	5.11	3.13	0.515	0.526	0.021 (1)	0.000	0.016
	Exotic adults	193	119	106.49	8.44	3.79	0.513	0.554	0.073 (2)	0.000	0.029
	Seeds	400	74	16.36	7.22	3.05	0.465	0.504	0.077 (1)	0.000	0.026
	Recruits‐R1	217	56	21.81	7.67	3.13	0.478	0.515	0.073 (4)	0.015	0.032
	Recruits‐R2	41	21	15.20	5.44	3.49	0.460	0.518	0.114 (0)	0.000	0.038

*n*, number of collected samples; *nh*, number of chloroplast haplotypes; *nh*_*e*_, effective number of chloroplast haplotypes; *na*, number of nSSR alleles; *na*_*e*_, effective number of nSSR alleles; *H*_*o*_, observed heterozygosity for nSSRs; *H*_*e*_, expected heterozygosity for nSSRs; *F*_IS_, inbreeding coefficient (number of nSSR loci in HW disequilibrium after Bonferroni correction between brackets); $F_{\mathrm{IS}}^{\prime }$, inbreeding coefficient jointly estimated with null allele frequencies; *f*(null), average null allele frequency across nSSR loci.

### Introgression estimates across sequential offspring samples

The estimated male gametic introgression rate from exotic plantations into the native *P. pinaster* population was low but significant (${\hat{m}}_{m}$ = 0.023; 95% CI: 0.011–0.041) at the seed stage (before seed dispersal), reached a zero value immediately after the summer seed dispersal period (R1 seedling sample), remained zero in the postwinter (R2) seedling sample, and increased to a higher but nonsignificant value of ${\hat{m}}_{m}$ = 0.053 in the last (and small) sequential seedling sample (R3) collected during the following summer (Table 3). Estimates of zygotic (${\hat{m}}_{z}$) and female gametic (${\hat{m}}_{f}$) introgression rates were not significantly different from zero in any of the *P. pinaster* seedling samples. Randomization and likelihood‐ratio tests indicated that none of the observed differences in introgression parameters among offspring samples of this species were significant (Table 3 and Supporting Information Table S5). Differences in ${\hat{m}}_{m}$ between seeds before dispersal and all seedlings pooled (R1 + R2 + R3) were not significant either (results not shown).

**Table 3:** Gene flow rate estimates from exotic plantations into the seed crop and naturally established recruitment of native *Pinus pinaster* at Fuencaliente and *P. sylvestris* at Sierra Nevada National Park

Species	Sample	${\hat{m}}_{m}$ (95% CI)	${\hat{m}}_{z}$ (95% CI)	${\hat{m}}_{f}$ (95% CI)
*P. pinaster*	Seeds	0.023 (0.011–0.041)		
	Recruits‐R1	0.000 (0.000–0.046)	0.000 (0.000–0.022)	0.016 (0.000–0.088)
	Recruits‐R2	0.000 (0.000–0.033)	0.000 (0.000–0.037)	0.007 (0.000–0.101)
	Recruits‐R3	0.053 (0.000–0.157)	0.000 (0.000–0.076)	0.000 (0.000–0.119)
*P. sylvestris*	Seeds	0.152 (0.116–0.194)		
	Recruits‐R1	0.087 (0.030–0.162)	0.064 (0.012–0.134)	0.000 (0.000–0.029)
	Recruits‐R2	0.078 (0.000–0.234)	0.019 (0.000–0.160)	0.000 (0.000–0.104)

${\hat{m}}_{m}$, ${\hat{m}}_{z}$, and$\;\;{\hat{m}}_{f}$, estimated male gametic, zygotic, and female gametic exotic gene flow rates, respectively; 95% confidence intervals (CI) computed using the profile‐likelihood method; Recruits‐R1, Recruits‐R2, Recruits‐R3, temporally sequential recruit (seedling) samples; permutations and likelihood‐ratio tests indicated that none of the three gene flow components exhibited significant differences across temporal samples (after multiple‐test corrections, see Supporting Information Tables S5 and S6).

For *P. sylvestris*, estimated male gametic introgression showed a maximum of ${\hat{m}}_{m}$ = 0.152 (95% CI: 0.116–0.194) before seed dispersal, dropping to ${\hat{m}}_{m}$ = 0.087 (95% CI: 0.030–0.162) immediately after the spring germination period (R1 sample), and showing a similar but nonsignificant value (${\hat{m}}_{m}$ = 0.078; 95% CI: 0.000–0.234) among the few seedlings found alive in the peak of the following summer (R2 sample) (Table 3). The decreasing trend in ${\hat{m}}_{m}$ was not significant, however, according to randomization and likelihood‐ratio tests (Supporting Information Table S6). Differences in ${\hat{m}}_{m}$ between seeds (before seed dispersal) and all seedlings pooled (R1 + R2) were not significant either (results not shown). Zygotic introgression rates from the plantations also exhibited a decreasing trend among established *P. sylvestris* seedlings, from a high of ${\hat{m}}_{z}$ = 0.064 (95% CI: 0.012–0.134) for R1 to a nonsignificant low of 0.019 in R2, but their difference was not significantly different from zero (Table 3 and Supporting Information Table S6). Estimates of female gametic introgression were zero in both *P. sylvestris* seedling samples.

## Discussion

We assessed, to our knowledge for the first time in the wild, potential early fitness consequences of gene flow from exotic tree plantations into native conspecific stands. We found significant proportions of exotic male gametes at the seed stage in the two studied relicts, which, subsequent to seed dispersal and seedling establishment, became nonsignificant in *Pinus pinaster* and dropped by half in *P. sylvestris*. Zygotic gene flow from the exotic plantations was absent in *P. pinaster*, while it was significant among early *P. sylvestris* recruits, decreasing throughout seedling establishment. Estimates of exotic female gametic gene flow were not significant in either of the two species. None of the differences in estimated exotic gene flow rates across temporally sequential samples were statistically significant, after accounting for multiple testing; we thus could not reject the null hypothesis of invariant early viability (survival rate) across parental cross types for either of the two species considered.

### Early fitness consequences of exotic gene flow

Our results are consistent with two alternative explanations: Either natural selection did not determine actual variation in postdispersal survival rates across parental cross types in the wild during the period of study (with observed nonsignificant variation being due to estimation error and stochastic mortality), or it actually did determine variation, but we failed to achieve statistical significance in our sequential sample comparison. Regarding the first possible explanation, equal expectations for the survival rates of native, exotic, and hybrid seedlings would imply an absence of local adaptation of native individuals at early stages of development. This would go against our initial working hypothesis, which we based on the more xeric conditions under which the relicts have probably evolved, relative to the most likely original habitats of the exotic plantations (Ramírez‐Valiente and Robledo‐Arnuncio 2014), and on the strong selective pressures operating during early stages of recruitment (Petit and Hampe 2006). Equal survival rates for different parental origins could alternatively be explained by harsh environmental conditions during the study period precluding the expression of some degree of actual adaptive genetic differentiation at early fitness traits. Indeed, an unambiguous fact is that the two recruitment cohorts under study approached total mortality at the end of the sampling season, demonstrating that, even if there were some actual variation among cross‐types in the speed of mortality (which we did not detect), parental origin ultimately did not influence final seedling viability. The question remains whether such influence would be exerted under milder environmental conditions, allowing at least some successful regeneration. Recruitment of long‐living tree species may depend disproportionately on rare years that have the right combination of favorable (mostly summer) conditions (Jackson et al. 2009). This would imply that ecologically relevant differentiation in early fitness traits among cross types might remain hidden unless assessed under such suitable conditions, an important caveat to consider in future studies.

The second possible explanation behind the nonrejection of our null hypothesis is that there was actual variation in seedling survival rates across parental types during the year of study, at least in the speed of mortality of *P. sylvestris* recruits, but we failed to achieve statistical significance in our tests. Although obviously our data does not statistically support this possibility, it is consistent with the (nonsignificant) decreasing trend in both male gametic and zygotic gene flow rates for *P. sylvestris*, and with the low statistical power to detect cross‐sample variation suggested by wide confidence intervals relative to the magnitude of estimates, especially for the last and smallest seedling samples (Table 3). This possibility is also consistent with results from previous studies in the same *P. sylvestris* population, which have shown (i) average post‐seed dispersal exotic male gametic introgression rates among 0‐ to 30‐year‐old recruits that were fairly similar to the ones found in our contemporary seedling samples (${\hat{m}}_{m}$ = 0.06 in Unger et al. 2014 versus ${\hat{m}}_{m}$ = 0.08–0.09 here, Table 3), but (ii) comparatively higher predispersal values at the seed stage during a single recent pollination season (${\hat{m}}_{m}$ = 0.39 in Robledo‐Arnuncio et al. 2009). Moreover, results from greenhouse experiments testing the same parental cross types considered here have shown significantly faster mortality for *P. sylvestris* hybrid and exotic seedlings under drought treatments (J.A. Ramírez‐Valiente and J.J. Robledo Arnuncio, unpublished data), also consistent with the hypothesis of actual variation in viability rates. In the case of *P. pinaster*, estimates of exotic gene flow were fairly low and only significantly different from zero before seed dispersal, suggesting low levels of actual introgression. The latter scenario complicates any inference about fitness consequences of introgression using our approach and precludes meaningful speculation about the small and nonsignificant differences observed across samples. This complication is aggravated by the fact that exotic gene flow tends to be somewhat overestimated for *P. pinaster* when it is actually absent (Supporting Information Annex S2). For *P. sylvestris*, by contrast, the model would accurately reflect the eventual absence of exotic gene flow, while tending to slight underestimation when it is actually positive, supporting our positive estimates for this species (Supporting Information Annex S2).

### Methodological challenges

Our results illustrate both the potential advantages and the challenges of short‐term longitudinal field studies to assess early fitness components of trees in the wild. The main advantage of the approach employed here is that it allowed us to test the null hypothesis of invariant viability of recruits of different paternal and maternal origins without costly controlled pollinations and without the potentially confounding interference of experimental manipulation (e.g., artificial environmental conditions during seed germination and seedling establishment, and reduced number of assayed parental genotypes). Moreover, the broad spatial scale of analysis, and therefore the range of micro‐environmental conditions and genotypic parental contributions of sampled recruits, approached those of the entirety of the target natural populations. The main drawbacks of our approach, shared by most studies assessing seedling establishment in the wild, are extremely high mortality (a biological contingency) compromising sample sizes required to achieve sufficient statistical power for hypothesis testing, and the need of temporal replication to account for strong interannual environmental variability. We conducted intensive field searches, collecting most (for intermediate samples) or all (for final samples) seedlings found, but still, looking retrospectively, it would probably have been more profitable in terms of statistical power to invest further sampling efforts in the first weeks immediately after seed dispersal, maybe reducing the total length of the sampling period and increasing the reference adult genotypic samples (Robledo‐Arnuncio 2012). This illustrates that designing an optimal sampling strategy for a detailed introgression study depends strongly on the biological setting, including interannual variance in dispersal and environmental parameters.

More generally, some of the assumptions behind our genetic and field approaches should be kept in mind for potential applications to other populations and plant species. We assumed that all possible parental populations were sampled, an assumption that was safe for our two isolated relict–plantation pairs. However, in scenarios with more populations or more continuously distributed species, approximating this assumption would require increased sampling requirements and would imply complicated admixture inference. We also assumed implicitly that different cross types exhibited similar seedling emergence phenology; that is, we assumed that the proportion of different cross types in the sequential seedling samples was influenced by the initial parental structure of the seed rain and the subsequent (selective or stochastic) mortality, but not by potential asynchronies in germination timing among crosses. Greenhouse experiments have shown synchronous germination for the same *P. sylvestris* cross types considered here (J.A. Ramírez‐Valiente and J.J. Robledo‐Arnuncio, unpublished data). Additionally, although we lack analogous data for our *P. pinaster* populations, field studies with other Iberian populations of the species have not found any significant difference in temporal patterns of emergence (Vizcaíno‐Palomar et al. 2014). This might not be the case for populations of other species, however. Caution should also be exercised if, unlike our two pine species (Ferrandis et al. 1996; Castro et al. 2005), the study taxon has a significant soil seed bank that has contributed to the collected contemporary seedling samples. If such a seed bank contribution is not taken into consideration, potentially different exotic gene flow rates during older reproductive seasons might confound the comparison of seed (before dispersal) versus seedling introgression rates.

Finally, it should be noted that had we detected statistically significant variation in estimated mortality rates for different cross types (i.e., not attributable to estimation error), it would still have been necessary to account for natural stochastic (genotype‐independent) mortality before ascribing the observed variation in viability to potential selective differences (e.g., Gompert et al. 2014). Random mortality appears to be responsible for a substantial proportion of observed variation in early survival rates among maternal families and populations of trees in the wild (Vizcaíno‐Palomar et al. 2014), which will probably also be the case for observed viability differences across intraspecific hybrid categories. This represents a big challenge when looking for potentially weak selective differentials in field studies. Given that we did not achieve statistical significance in our temporal sample comparison, however, it was meaningless to proceed to contrast the random mortality hypothesis in our particular cases.

### Management implications

Regional forest managers are implementing several conservation genetic strategies for the two pine relicts studied here, including *ex situ* germplasm collections and *in situ* population reinforcement, both based on open‐pollinated seed collections from native mothers. Results from this study can inform such strategies with respect to both the extent of the exposure of native stands to exotic genes and the potential adaptive consequences of such exposure. In terms of exposure assessment, our results indicate that the contemporary inflow of exotic pollen into the seed crop of native *P. sylvestris* mothers may fluctuate between years but is sustained at a rather elevated level (estimates of ${\hat{m}}_{m}$ = 0.39 in Robledo‐Arnuncio et al. 2009 and ${\hat{m}}_{m}$ = 0.15 in this study, Table 3). Our results have also revealed that the corresponding *m*_*m*_‐estimate at the seed stage is lower for the relatively more isolated *P. pinaster* relict than for *P. sylvestris*, but still significant. Therefore, if the goal were to preserve native gene pools strictly free of exotic genes, then both *ex situ* collections and *in situ* reinforcements would ideally be based on clonal reproductive material or controlled‐pollinated seeds from native parents. On the other hand, our results reveal seed dispersal as an incipient source of exotic gene flow into the study *P. sylvestris* relict, with a significant 6% estimate of contemporary zygotic gene flow that contrasts with the zero value obtained among older saplings established in previous decades (Unger et al. 2014). Future monitoring activities should verify whether the pressure of exotic seeds might be increasing as the relatively young plantations age.

Regarding the potential adaptive consequences of exotic gene flow, our results do not reveal significant differences in early fitness among local, exotic, and F1 hybrid seedlings in the wild, which would suggest that forest managers should not expect an adaptive impact (either negative or positive) of initial steps of exotic introgression within the pine relicts. It could further be argued that, given the apparent absence of outbreeding depression, exotic gene flow could be regarded as a positive process in the long term, as it might eventually enhance adaptation of the small relicts to future environmental changes via increased genetic variance available for selection (Alleaume‐Benharira et al. 2006; Lopez et al. 2008; Duputié et al. 2012). Whether this will actually be the case is uncertain, however, as recombination in F2 hybrids and later recombinants might result in outbreeding depression at later stages of exotic introgression (Fenster and Galloway 2000). Additionally, further field experiments are required to establish potential early fitness variation among cross types under natural environmental conditions allowing successful recruitment. We believe that a reasonable management strategy, trading off between precaution and cost, could combine *ex situ* preservation of native germplasm with periodic *in situ* monitoring of the level of exotic gene flow and of fitness variation among naturally established recruits of local, exotic, and hybrid origin (especially under suitable conditions for recruitment). More costly and extreme measures have been proposed, such as gradually replacing all natural recruitment within the relicts (or even the exotic plantations themselves) with local individuals, but we suggest such measures be reserved for the hypothetical cases of genetic monitoring revealing deleterious effects of exotic gene flow, or for situations in which there are societal demands for a strict preservation of the relicts’ gene pools.

## Data archiving statement

Data available from the Dryad Digital Repository: http://dx.doi.org/10.5061/dryad.13bd2.

## Supporting information


**Annex S1.** Estimation of genotypic probabilities.
**Annex S2.** Validation tests of inference model and molecular markers.
**Table S1.** Chloroplast haplotype frequencies for *Pinus pinaster* adults and offspring.
**Table S2.** Chloroplast haplotype frequencies for *Pinus sylvestris* adults and offspring.
**Table S3.** Allelic frequencies for nSSR loci in *Pinus pinaster*.
**Table S4.** Allelic frequencies for nSSR loci in *Pinus sylvestris*.
**Table S5.** Statistical significance of gene flow rate comparisons for *Pinus pinaster*.
**Table S6.** Statistical significance of gene flow rate comparisons for *Pinus sylvestris*.
