# Supplementary material for: Assessing early fitness consequences of exotic gene flow in the wild: a field study with Iberian pine relicts
Source: Evol Appl. 2016 Jan 9;9(2):367–80. doi: 10.1111/eva.12333 (PMC4721076; doi:10.1111/eva.12333)
Supplement: Supplementary file 1 — Annex S1. Estimation of genotypic probabilities. Annex S2. Validation tests of inference model and molecular markers. Table S1. Chloroplast haplotype frequencies for Pinus pinaster adults and offspring. Table S2. Chloroplast haplotype frequencies for Pinus sylvestris adults and offspring. Table S3. Allelic frequencies for nSSR loci in Pinus pinaster. Table S4. Allelic frequencies for nSSR loci in Pinus sylvestris. Table S5. Statistical significance of gene flow rate comparisons for Pinus pinaster. Table S6. Statistical significance of gene flow rate comparisons for Pinus sylvestris. [file EVA-9-367-s001.doc]

**Supporting Information**

**Assessing Early Fitness Consequences of Exotic Gene Flow in The Wild: A Field Study with Iberian Pine Relicts**

**Annex S1.** Estimation of genotypic probabilities. 2

**Annex S2.** Validation tests of inference model and molecular markers. 5

**Table S1.** Chloroplast haplotype frequencies for *Pinus pinaster* adults and offspring. 10

**Table S2.** Chloroplast haplotype frequencies for *Pinus sylvestris* adults and offspring. 13

**Table S3.** Allelic frequencies for nSSR loci in *Pinus pinaster*. 19

**Table S4.** Allelic frequencies for nSSR loci in *Pinus sylvestris*. 23

**Table S5.** Statistical significance of gene flow rate comparisons for *Pinus pinaster*. 26

**Table S6.** Statistical significance of gene flow rate comparisons for *Pinus sylvestris*. 27

**Literature cited.** 27

**Annex S1.** Estimation of genotypic probabilities.

We denoted *G_R_* the multilocus genotype of a sampled seedling, comprising its diploid genotype at *L* nSSR loci ($G_{R}^{nu}$) and its cpSSR haplotype ($G_{R}^{cp}$), with the latter defined as the observed combination of variants at all analysed cpSSR loci. We further defined $\Pr_{PN}(G_{R}|n_{P},n_{N})$, $\Pr_{PP}(G_{R}|n_{P})$, $\Pr_{NP}(G_{R}|n_{P},n_{N})$ and $\Pr_{NN}(G_{R}|n_{N})$ as the probabilities that a seedling has genotype *G_R_* if it was born to a native mother pollinated by an exotic father, two exotic parents, an exotic mother pollinated by a native father, or two native parents, respectively, given the reference adult genotypic frequencies estimated from the *n_P_* exotic and *n_N_* native adults. Following Unger et al. (2014), these combined chloroplast-nuclear multilocus genotypic probabilities correspond to the product of the independent genotypic probabilities for $G_{R}^{cp}$ and $G_{R}^{nu}$, which can be written as

$\Pr_{ij}(G_{R}|n_{i},n_{j})=\Pr_{i}(G_{R}^{cp}|n_{i})\cdot\prod_{l=1}^{L} \Pr_{ij}(G_{R,l}^{nu}|n_{i},n_{j})$, (S1)

assuming that the *L* nuclear loci are unlinked, and where *i* and *j* denote the paternal and maternal populations of origin, respectively.

Assuming Hardy-Weinberg equilibrium, the single-locus genotypic probabilities for the nuclear loci, $\Pr_{ij}(G_{R,l}^{nu}|n_{i},n_{j})$, are typically estimated in either of two ways for the purpose of genetic assignment (Rannala & Mountain 1997). The obvious approach is to estimate them directly as

$\Pr_{ij}(G_{R,l}^{nu}|n_{i},n_{j})=\left\{\begin{matrix} \frac{n_{ail}^{2}}{n_{il}^{2}} \text{if} i=j \text{and} G_{R,l}^{nu}=aa \\ \frac{2n_{ail}n_{bil}}{n_{il}^{2}} \text{if} i=j \text{and} G_{R,l}^{nu}=ab \\ \frac{n_{ail}n_{ajl}}{n_{il}n_{jl}} \text{if} i\neq j \text{and} G_{R,l}^{nu}=aa \\ \frac{n_{ail}n_{bjl}+n_{bil}n_{ajl}}{n_{il}n_{jl}} \text{if} i\neq j \text{and} G_{R,l}^{nu}=ab \end{matrix}\right.$ (S2)

depending on whether $G_{R,l}^{nu}$ is homozygous (*aa*) or heterozygous (*ab*), and on whether the paternal and maternal gametic phases originated from the same (*i* = *j*) or different populations (*i* ≠ *j*), and where *n_ail_* is the observed number of copies of allele *a* at the *l*-th locus in the adult sample from the *i-*th population, and *n_il_* is the adult sample size (number of chromosomes) at the *l*-th locus for the *i-*th population. Alternatively, posterior genotypic probabilities $\Pr_{ij}^{'}(G_{R,l}^{nu}|n_{i},n_{j})$ can be estimated under the prior assumption that the total number of alleles at each locus for each population is identical to the total number of alleles for that locus (*k_l_*) in the pooled sample of adults, following the Bayesian approach of Rannala & Mountain (1997):

$\mathrm{P}\mathrm{r}'_{ij}(G_{R,l}^{nu}|n_{i},n_{j})=\left\{\begin{matrix} \frac{(n_{ail}+1/k_{l}+1)(n_{ail}+1/k_{l})}{(n_{il}+2)(n_{il}+1)} \text{if} i=j \text{and} G_{R,l}^{nu}=aa \\ \frac{2(n_{ail}+1/k_{l})(n_{bil}+1/k_{l})}{(n_{il}+2)(n_{il}+1)} \text{if} i=j \text{and} G_{R,l}^{nu}=ab \\ \frac{(n_{ail}+1/k_{l})(n_{ajl}+1/k_{l})}{(n_{il}+1)(n_{jl}+1)} \text{if} i\neq j \text{and} G_{R,l}^{nu}=aa \\ \frac{(n_{ail}+1/k_{l})(n_{bjl}+1/k_{l})+(n_{bil}+1/k_{l})(n_{ajl}+1/k_{l})}{(n_{il}+1)(n_{jl}+1)} \text{if} i\neq j \text{and} G_{R,l}^{nu}=ab \end{matrix}\right.$ (S3)

Analogously, chloroplast haplotypic frequencies can be calculated either directly as

$\Pr_{i}(G_{R}^{cp}|n_{i})=n_{G_{R}^{cp}}/n_{i}$, (S4)

where $n_{G_{R}^{cp}}$ is the observed count of the $G_{R}^{cp}$ haplotype among the *n_i_* adult trees sampled from population *i*, or using the Bayesian approach as

$\Pr_{i}^{'}(G_{R}^{cp}|n_{i})=(n_{G_{R}^{cp}}+1/k_{cp})/(n_{i}+1)$, (S5)

where *k_cp_* is the total number of cp-haplotypes in the pooled sample of adults.

The Bayesian approach is meant to deal with potential biases derived from undetected alleles (or cp-haplotypes) in small adult samples. Given the sample sizes and observed counts of nSSR alleles and cp-haplotypes for each of the two species, and using the numerical simulation scheme described in Unger et al. (2014), we checked which of the two procedures resulted in more accurate gene flow rate estimates and better coverage of associated confidence intervals. We found that the two approaches yielded virtually identical results in the case of *Pinus sylvestris*, while in *P. pinaster* the Bayesian method provided slightly less biased and less variable gene flow estimates with better actual confidence interval coverage (results not shown). Therefore, we used eqns (S2) and (S4) for *P. sylvestris* and eqns (S3) and (S5) for *P. pinaster*.

**Annex S2.** Validation tests of inference model and molecular markers.

*Methods*

Unger et al. (2014) conducted a numerical simulation analysis of the statistical performance of the same maximum-likelihood method used in the present study to estimate intraspecific exotic gene flow components. Their results, suggesting generally low bias and mean-square error for estimated gene flow rates, as well as confidence interval coverage close to nominal values, hold for our study as well, since their analysis was based on the empirical population frequencies of nSSR alleles and cpSSR haplotypes at the same set of loci, and at the same native stands and non-local plantations, considered in the present case. Unger et al. (2014) used simulated offspring genotypes of different parental origins to test the method, generating them via appropriate stochastic Hardy-Weinberg draws from the corresponding empirical population allelic frequency distributions (see their Appendix S2 in Supporting Information). We further test the method here obviating the Hardy-Weinberg assumption, by using a cross-validation approach on real adult genotypes of known origin. In particular, we considered the empirical sample of native adult genotypes (*n*_N_ = 200 for *Pinus sylvestris* and *n*_N_ = 100 for *P. pinaster*) and exotic adult genotypes (*n*_P_ = 185 for *P. sylvestris* and *n*_P_ = 136 for *P. pinaster*), and used the four following algorithms for each of the two species:

(A) We first followed these steps to assess the method’s ability to estimate accurately the proportion of native genotypes in a pure native sample (in which *m_m_* = *m_z_* = *m_f_* = 0):

(1A) Randomly partition the total *n*_N_ sample into a *test subset* of 50 genotypes and a reduced *reference subset* of *n*_N_ – 50 genotypes.

(2A) Estimate reference native adult population allelic frequencies using the reduced *reference subset*.

(3A) Use eqn 1 (in main text) to estimate the proportion of native individuals (as 1 – *m_m_* – *m_z_* – *m_f_*) in the *test subset* (considering the full *n*_P_ sample to estimate exotic adult population allelic frequencies).

We repeated this procedure *N* = 1,000 times to obtain the distribution of the estimated proportion of native individuals in a pure native sample, used in turn to estimate the expected bias and expected root mean square error (*RMSE*) as:

(B) We then assessed the method’s ability to estimate the proportion of exotic genotypes in a pure exotic sample (in which *m_z_* = 1) as follows:

(1B) Randomly partition the total *n*_P_ sample into a *test subset* of 50 genotypes and a reduced *reference subset* of *n*_P_ – 50 genotypes.

(2B) Estimate reference exotic adult population allelic frequencies using the reduced *reference subset*.

(3B) Use eqn 1 (in main text) to estimate the proportion of exotic individuals (as *m_z_*) in the *test subset* (considering the full *n*_N_ sample to estimate native adult population allelic frequencies).

Repeating this procedure *N* = 1,000 times, the expected bias and *RMSE* of the estimated proportion of exotic individuals in a pure exotic sample is calculated as:

(C) We then generated *in-silico* Mendelian crosses between real native mothers and real exotic fathers to test the method’s ability to estimate the proportion of male gametic immigrants in a sample entirely composed of them (i.e., *m_m_* = 1):

(1C) Randomly partition the total *n*_N_ sample into a *test subset* of 50 genotypes and a reduced *reference subset* of *n*_N_ – 50 genotypes.

(2C) Randomly partition the total *n*_P_ sample into a *test subset* of 50 genotypes and a reduced *reference subset* of *n*_P_ – 50 genotypes.

(3C) Repeat this step 50 times to obtain a *simulated offspring sample* of size 50: randomly select a native mother and an exotic father from the respective *test subsets* and generate an offspring genotype through Mendelian segregation of nSSR alleles and paternal inheritance of cpSSR haplotype.

(4C) Estimate reference native and exotic adult population allelic frequencies using the respective reduced *reference subsets.*

(5C) Use eqn 1 (in main text) to estimate the proportion of male gametic gene flow (*m_m_*) in the *simulated offspring sample*.

Repeating this procedure *N* = 1,000 times, the expected bias and *RMSE* of the estimated proportion of male gametic immigrants ($\hat{m}_{m}$) in a sample of 50 offspring with *m_m_* = 1 is calculated as:

(D) Finally, we generated *in-silico* Mendelian crosses between real exotic mothers and real native fathers to test the method’s ability to estimate the proportion of female gametic immigrants in a sample entirely composed of them (i.e., *m_f_* = 1):

(1D) Randomly partition the total *n*_N_ sample into a *test subset* of 50 genotypes and a reduced *reference subset* of *n*_N_ – 50 genotypes.

(2D) Randomly partition the total *n*_P_ sample into a *test subset* of 50 genotypes and a reduced *reference subset* of *n*_P_ – 50 genotypes.

(3D) Repeat this step 50 times to obtain a *simulated offspring sample* of size 50: randomly select an exotic mother and a native father from the respective *test subsets* and generate an offspring genotype through Mendelian segregation of nSSR alleles and paternal inheritance of cpSSR haplotype.

(4D) Estimate reference native and exotic adult population allelic frequencies using the respective reduced *reference subsets.*

(5D) Use eqn 1 (in main text) to estimate the proportion of female gametic gene flow (*m_f_*) in the *simulated offspring sample*.

Repeating this procedure *N* = 1,000 times, the expected bias and *RMSE* of the estimated proportion of male gametic immigrants ($\hat{m}_{f}$) in a sample of 50 offspring with *m_m_* = 1 is calculated as:

*Results*

Results obtained from each of the four analyses (A to D) were as follows:

|  | (A) *Pure native* | |  | (B) *Pure exotic* | |  | (C) *Native x exotic* | |  | (D) *Exotic x native* | |
| --- | --- | --- | --- | --- | --- | --- | --- | --- | --- | --- | --- |
| Species | Bias | *RMSE* |  | Bias | *RMSE* |  | Bias | *RMSE* |  | Bias | *RMSE* |
| *P. pinaster* | -0.080 | 0.100 |  | -0.018 | 0.056 |  | -0.087 | 0.141 |  | -0.076 | 0.121 |
| *P. sylvestris* | -0.011 | 0.034 |  | -0.077 | 0.102 |  | -0.086 | 0.124 |  | -0.069 | 0.104 |

For *P. sylvestris*, applying the method to a sample entirely composed of native individuals yielded very lowly negatively biased (–1.1%) and fairly accurate (*RMSE* of 3.4%) estimates of the proportion of pure native genotypes. Estimates of *m_z_*, *m_m_* and *m_f_* in samples entirely composed of, respectively, pure exotic, native x exotic or exotic x native *P. sylvestris* genotypes suffered somewhat larger negative biases (ranging from 7–9%) and *RMSE* (ranging from 10-12%). These results are fully consistent with those in Unger et al. (2014), and suggest that the model, when applied to the study populations of *P. sylvestris*, does not overestimate exotic gene flow when it is absent, while it slightly underestimates all its three components when it’s present.

For *P. pinaster*, the method somewhat underestimated (–8.0%) the proportion of native genotypes in samples entirely composed of pure native genotypes, as well as *m_m_* and *m_f_* in samples comprising native x exotic or exotic x native genotypes, respectively, while it estimated fairly accurately *m_z_* (–1.8% bias, 5.6% *RMSE*) in purely exotic samples. Our results for *P. pinaster* are also consistent with Unger et al. (2014), suggesting that the model tend to yield low but positive exotic gene flow estimates when exotic gene flow is actually absent, while it tends to underestimate *m_m_* and *m_f_* (and to a lesser extent *m_z_*) when their actual values are positive.

**Table S1.** Chloroplast haplotype frequencies for *Pinus pinaster* adults and offspring.

| Haplotype | | |  | Sample | | | | | | | | | | |
| --- | --- | --- | --- | --- | --- | --- | --- | --- | --- | --- | --- | --- | --- | --- |
|  |  |  |  | Adults | | |  | Seeds |  | Recruits | | | | |
| Code |  | Size variants |  | Native |  | Exotic |  |  |  | R1 |  | R2 |  | R3 |
| H1 |  | 145-145-117-144-164-80 |  | 0.4600 |  | 0.0519 |  | 0.4376 |  | 0.5052 |  | 0.5926 |  | 0.6444 |
| H2 |  | 140-145-116-144-165-79 |  | 0.1400 |  | 0 |  | 0.1453 |  | 0.0928 |  | 0.1019 |  | 0.1111 |
| H3 |  | 140-145-116-143-165-79 |  | 0.1200 |  | 0 |  | 0.1153 |  | 0.1856 |  | 0.1111 |  | 0.0667 |
| H4 |  | 140-147-114-144-163-80 |  | 0.0800 |  | 0 |  | 0.1343 |  | 0.0619 |  | 0.0741 |  | 0.0222 |
| H5 |  | 145-146-115-144-163-78 |  | 0.0400 |  | 0.0074 |  | 0.0284 |  | 0.0103 |  | 0.0370 |  | 0 |
| H6 |  | 145-145-114-144-163-79 |  | 0.0300 |  | 0.0074 |  | 0.0348 |  | 0.0412 |  | 0.0370 |  | 0.0889 |
| H7 |  | 145-145-114-145-163-79 |  | 0.0300 |  | 0.0074 |  | 0 |  | 0 |  | 0 |  | 0 |
| H8 |  | 145-145-115-144-163-79 |  | 0.0200 |  | 0.0889 |  | 0.0111 |  | 0.0206 |  | 0.0185 |  | 0 |
| H9 |  | 145-145-115-144-163-80 |  | 0.0200 |  | 0.0222 |  | 0.0016 |  | 0.0103 |  | 0 |  | 0 |
| H10 |  | 145-145-114-144-161-80 |  | 0.0100 |  | 0.0074 |  | 0.0016 |  | 0.0103 |  | 0 |  | 0 |
| H11 |  | 145-144-115-144-163-81 |  | 0.0100 |  | 0.0296 |  | 0.0237 |  | 0.0103 |  | 0.0185 |  | 0 |
| H12 |  | 145-145-114-144-163-78 |  | 0.0100 |  | 0.0074 |  | 0.0079 |  | 0.0103 |  | 0 |  | 0.0222 |
| H13 |  | 145-146-114-144-162-81 |  | 0.0100 |  | 0.0370 |  | 0.0032 |  | 0 |  | 0 |  | 0 |
| H14 |  | 145-145-115-144-162-80 |  | 0.0100 |  | 0.0963 |  | 0.0016 |  | 0 |  | 0 |  | 0 |
| H15 |  | 145-145-115-145-162-81 |  | 0.0100 |  | 0.0296 |  | 0.0016 |  | 0 |  | 0 |  | 0 |
| H16 |  | 145-145-114-144-163-80 |  | 0 |  | 0.0222 |  | 0 |  | 0 |  | 0 |  | 0 |
| H17 |  | 145-145-115-144-163-73 |  | 0 |  | 0.0074 |  | 0 |  | 0 |  | 0 |  | 0 |
| H18 |  | 145-145-115-144-162-73 |  | 0 |  | 0.0148 |  | 0 |  | 0 |  | 0 |  | 0 |
| H19 |  | 145-143-115-145-164-86 |  | 0 |  | 0.0222 |  | 0 |  | 0 |  | 0 |  | 0 |
| H20 |  | 145-145-115-145-163-79 |  | 0 |  | 0.0074 |  | 0 |  | 0 |  | 0 |  | 0 |
| H21 |  | 145-145-117-144-163-80 |  | 0 |  | 0.0148 |  | 0.0032 |  | 0 |  | 0 |  | 0 |
| H22 |  | 145-144-115-144-163-82 |  | 0 |  | 0.0148 |  | 0 |  | 0 |  | 0 |  | 0 |
| H23 |  | 145-145-116-144-162-81 |  | 0 |  | 0.0074 |  | 0 |  | 0 |  | 0 |  | 0 |
| H24 |  | 145-145-115-147-163-83 |  | 0 |  | 0.0296 |  | 0 |  | 0 |  | 0 |  | 0 |
| H25 |  | 145-143-116-145-163-81 |  | 0 |  | 0.0074 |  | 0 |  | 0 |  | 0 |  | 0 |
| H26 |  | 140-146-115-144-163-78 |  | 0 |  | 0.0074 |  | 0 |  | 0 |  | 0 |  | 0 |
| H27 |  | 145-145-115-144-162-81 |  | 0 |  | 0.0593 |  | 0.0016 |  | 0 |  | 0 |  | 0 |
| H28 |  | 145-145-118-144-163-80 |  | 0 |  | 0.0148 |  | 0 |  | 0 |  | 0 |  | 0 |
| H29 |  | 145-145-116-144-162-80 |  | 0 |  | 0.0148 |  | 0 |  | 0 |  | 0 |  | 0 |
| H30 |  | 145-145-115-144-162-79 |  | 0 |  | 0.0296 |  | 0.0016 |  | 0 |  | 0 |  | 0 |
| H31 |  | 145-143-116-144-164-85 |  | 0 |  | 0.0074 |  | 0 |  | 0 |  | 0 |  | 0 |
| H32 |  | 140-145-116-145-163-80 |  | 0 |  | 0.0074 |  | 0 |  | 0 |  | 0 |  | 0 |
| H33 |  | 140-146-115-144-163-81 |  | 0 |  | 0.0222 |  | 0 |  | 0 |  | 0 |  | 0.0222 |
| H34 |  | 145-144-114-144-162-80 |  | 0 |  | 0.0074 |  | 0.0016 |  | 0 |  | 0 |  | 0 |

Size variants from Pt36480-Pt30204-Pt15169-Pt71936-Pt87268-Pt1254.

**Table S1 (continued).** Chloroplast haplotype frequencies for *Pinus pinaster* adults and offspring.

| Haplotype | | |  | Sample | | | | | | | | | | |
| --- | --- | --- | --- | --- | --- | --- | --- | --- | --- | --- | --- | --- | --- | --- |
|  |  |  |  | Adults | | |  | Seeds |  | Recruits | | | | |
| Code |  | Size variants |  | Native |  | Exotic |  |  |  | R1 |  | R2 |  | R3 |
| H35 |  | 145-145-114-144-162-80 |  | 0 |  | 0.0074 |  | 0 |  | 0 |  | 0 |  | 0 |
| H36 |  | 145-144-115-144-162-81 |  | 0 |  | 0.0593 |  | 0.0016 |  | 0 |  | 0 |  | 0 |
| H37 |  | 140-145-115-144-165-79 |  | 0 |  | 0.0074 |  | 0.0016 |  | 0 |  | 0 |  | 0 |
| H38 |  | 140-146-115-144-163-80 |  | 0 |  | 0.0074 |  | 0 |  | 0 |  | 0 |  | 0 |
| H39 |  | 145-145-115-144-163-81 |  | 0 |  | 0.0148 |  | 0.0016 |  | 0 |  | 0 |  | 0 |
| H40 |  | 140-145-115-145-163-80 |  | 0 |  | 0.0074 |  | 0 |  | 0 |  | 0 |  | 0 |
| H41 |  | 145-147-114-143-163-67 |  | 0 |  | 0.0074 |  | 0 |  | 0 |  | 0 |  | 0 |
| H42 |  | 140-146-115-145-164-79 |  | 0 |  | 0.0074 |  | 0 |  | 0 |  | 0 |  | 0 |
| H43 |  | 145-146-114-144-163-80 |  | 0 |  | 0.0074 |  | 0 |  | 0 |  | 0 |  | 0 |
| H44 |  | 145-144-115-144-163-80 |  | 0 |  | 0.0074 |  | 0.0016 |  | 0 |  | 0 |  | 0.0222 |
| H45 |  | 145-145-116-144-163-80 |  | 0 |  | 0.0148 |  | 0 |  | 0 |  | 0 |  | 0 |
| H46 |  | 145-143-116-144-164-79 |  | 0 |  | 0.0148 |  | 0 |  | 0 |  | 0 |  | 0 |
| H47 |  | 145-144-116-143-164-72 |  | 0 |  | 0.0074 |  | 0 |  | 0 |  | 0 |  | 0 |
| H48 |  | 145-144-115-144-163-79 |  | 0 |  | 0.0074 |  | 0 |  | 0 |  | 0 |  | 0 |
| H49 |  | 145-145-115-145-163-80 |  | 0 |  | 0.0074 |  | 0 |  | 0 |  | 0.0093 |  | 0 |
| H50 |  | 145-145-114-144-162-84 |  | 0 |  | 0.0074 |  | 0 |  | 0 |  | 0 |  | 0 |
| H51 |  | 145-145-115-144-163-82 |  | 0 |  | 0.0074 |  | 0 |  | 0 |  | 0 |  | 0 |
| H52 |  | 145-145-117-143-163-78 |  | 0 |  | 0.0074 |  | 0 |  | 0 |  | 0 |  | 0 |
| H53 |  | 145-146-114-144-163-79 |  | 0 |  | 0.0074 |  | 0.0016 |  | 0 |  | 0 |  | 0 |
| H54 |  | 145-146-113-144-162-80 |  | 0 |  | 0.0148 |  | 0 |  | 0 |  | 0 |  | 0 |
| H55 |  | 145-144-117-144-162-80 |  | 0 |  | 0.0074 |  | 0 |  | 0 |  | 0 |  | 0 |
| H56 |  | 145-144-115-145-164-78 |  | 0 |  | 0.0074 |  | 0 |  | 0 |  | 0 |  | 0 |
| H57 |  | 145-145-115-144-162-78 |  | 0 |  | 0.0074 |  | 0 |  | 0 |  | 0 |  | 0 |
| H58 |  | 140-146-115-144-163-79 |  | 0 |  | 0.0074 |  | 0 |  | 0 |  | 0 |  | 0 |
| H59 |  | 140-146-115-145-163-78 |  | 0 |  | 0.0074 |  | 0 |  | 0 |  | 0 |  | 0 |
| H60 |  | 145-143-116-145-164-79 |  | 0 |  | 0.0074 |  | 0 |  | 0 |  | 0 |  | 0 |
| H61 |  | 145-146-114-145-164-80 |  | 0 |  | 0.0074 |  | 0.0016 |  | 0 |  | 0 |  | 0 |
| H62 |  | 145-145-116-144-164-80 |  | 0 |  | 0.0074 |  | 0 |  | 0 |  | 0 |  | 0 |
| H63 |  | 140-144-116-143-165-79 |  | 0 |  | 0 |  | 0.0016 |  | 0 |  | 0 |  | 0 |
| H64 |  | 145-144-115-144-162-80 |  | 0 |  | 0 |  | 0.0016 |  | 0.0103 |  | 0 |  | 0 |

Size variants from Pt36480-Pt30204-Pt15169-Pt71936-Pt87268-Pt1254.

**Table S1 (continued).** Chloroplast haplotype frequencies for *Pinus pinaster* adults and offspring.

| Haplotype | | |  | Sample | | | | | | | | | | |
| --- | --- | --- | --- | --- | --- | --- | --- | --- | --- | --- | --- | --- | --- | --- |
|  |  |  |  | Adults | | |  | Seeds |  | Recruits | | | | |
| Code |  | Size variants |  | Native |  | Exotic |  |  |  | R1 |  | R2 |  | R3 |
| H65 |  | 145-145-114-144-162-81 |  | 0 |  | 0 |  | 0.0016 |  | 0 |  | 0 |  | 0 |
| H66 |  | 145-145-115-143-162-76 |  | 0 |  | 0 |  | 0.0016 |  | 0 |  | 0 |  | 0 |
| H67 |  | 140-145-117-144-162-81 |  | 0 |  | 0 |  | 0.0016 |  | 0 |  | 0 |  | 0 |
| H68 |  | 145-144-114-145-162-84 |  | 0 |  | 0 |  | 0.0079 |  | 0 |  | 0 |  | 0 |
| H69 |  | 140-146-114-144-163-79 |  | 0 |  | 0 |  | 0.0016 |  | 0 |  | 0 |  | 0 |
| H70 |  | 145-145-117-144-164-79 |  | 0 |  | 0 |  | 0.0016 |  | 0 |  | 0 |  | 0 |
| H71 |  | 140-144-115-144-165-79 |  | 0 |  | 0 |  | 0.0016 |  | 0 |  | 0 |  | 0 |
| H72 |  | 140-146-114-144-163-81 |  | 0 |  | 0 |  | 0.0016 |  | 0 |  | 0 |  | 0 |
| H73 |  | 145-145-115-146-163-81 |  | 0 |  | 0 |  | 0.0016 |  | 0 |  | 0 |  | 0 |
| H74 |  | 146-145-114-144-163-79 |  | 0 |  | 0 |  | 0.0047 |  | 0 |  | 0 |  | 0 |
| H75 |  | 145-145-115-144-164-79 |  | 0 |  | 0 |  | 0.0016 |  | 0.0103 |  | 0 |  | 0 |
| H76 |  | 145-145-115-146-162-82 |  | 0 |  | 0 |  | 0.0016 |  | 0 |  | 0 |  | 0 |
| H77 |  | 140-145-115-144-165-80 |  | 0 |  | 0 |  | 0.0016 |  | 0 |  | 0 |  | 0 |
| H78 |  | 140-146-114-144-163-80 |  | 0 |  | 0 |  | 0.0016 |  | 0 |  | 0 |  | 0 |
| H79 |  | 145-145-115-144-163-78 |  | 0 |  | 0 |  | 0 |  | 0.0103 |  | 0 |  | 0 |
| H80 |  | 145-146-116-144-162-81 |  | 0 |  | 0 |  | 0 |  | 0.0103 |  | 0 |  | 0 |

Size variants from Pt36480-Pt30204-Pt15169-Pt71936-Pt87268-Pt1254.

**Table S2.** Chloroplast haplotype frequencies for *Pinus sylvestris* adults and offspring.

| Haplotype | | |  | Sample | | | | | | | | |
| --- | --- | --- | --- | --- | --- | --- | --- | --- | --- | --- | --- | --- |
|  |  |  |  | Adults | | |  | Seeds |  | Recruits | | |
| Code |  | Size variants |  | Native |  | Exotic |  |  |  | R1 |  | R2 |
| H1 |  | 165-144-110-126-146-145-67 |  | 0.2041 |  | 0.0518 |  | 0.1125 |  | 0.1215 |  | 0.2195 |
| H2 |  | 165-144-110-126-146-142-68 |  | 0.1531 |  | 0.0155 |  | 0.1525 |  | 0.0841 |  | 0.0732 |
| H3 |  | 165-144-110-125-146-141-67 |  | 0.1378 |  | 0.0155 |  | 0.0850 |  | 0.0841 |  | 0.0488 |
| H4 |  | 166-144-110-125-147-145-66 |  | 0.1378 |  | 0 |  | 0.0850 |  | 0.0607 |  | 0.0732 |
| H5 |  | 165-144-110-126-144-141-67 |  | 0.0459 |  | 0 |  | 0.0225 |  | 0.0280 |  | 0 |
| H6 |  | 165-144-110-126-146-141-66 |  | 0.0459 |  | 0.0052 |  | 0.0675 |  | 0.0561 |  | 0.0244 |
| H7 |  | 165-144-110-127-145-143-67 |  | 0.0408 |  | 0.0104 |  | 0.0175 |  | 0.0140 |  | 0 |
| H8 |  | 165-144-110-125-146-146-67 |  | 0.0357 |  | 0.0104 |  | 0.0175 |  | 0.0234 |  | 0 |
| H9 |  | 165-144-109-125-148-144-67 |  | 0.0357 |  | 0 |  | 0.0175 |  | 0.0327 |  | 0 |
| H10 |  | 165-144-110-126-145-143-67 |  | 0.0357 |  | 0.0052 |  | 0.0250 |  | 0.0654 |  | 0.0732 |
| H11 |  | 165-144-109-126-146-144-67 |  | 0.0255 |  | 0 |  | 0.0275 |  | 0.0327 |  | 0 |
| H12 |  | 165-143-110-126-146-144-67 |  | 0.0255 |  | 0 |  | 0.0400 |  | 0.0187 |  | 0.0488 |
| H13 |  | 166-144-109-125-147-143-66 |  | 0.0153 |  | 0 |  | 0.0400 |  | 0.0467 |  | 0 |
| H14 |  | 165-144-110-126-148-143-67 |  | 0.0102 |  | 0 |  | 0.0150 |  | 0.0280 |  | 0.0244 |
| H15 |  | 166-144-110-125-146-145-68 |  | 0.0102 |  | 0 |  | 0.0250 |  | 0.0140 |  | 0.0244 |
| H16 |  | 165-144-110-127-145-142-67 |  | 0.0051 |  | 0 |  | 0 |  | 0 |  | 0 |
| H17 |  | 165-144-110-126-149-143-67 |  | 0.0051 |  | 0 |  | 0.0075 |  | 0.0140 |  | 0 |
| H18 |  | 166-144-110-125-147-142-67 |  | 0.0051 |  | 0.0052 |  | 0.0025 |  | 0 |  | 0 |
| H19 |  | 166-143-110-126-146-144-66 |  | 0.0051 |  | 0 |  | 0 |  | 0 |  | 0 |
| H20 |  | 166-144-110-125-147-144-67 |  | 0.0051 |  | 0 |  | 0 |  | 0 |  | 0 |
| H21 |  | 166-144-110-125-147-146-66 |  | 0.0051 |  | 0 |  | 0.0200 |  | 0.0327 |  | 0.0732 |
| H22 |  | 165-143-110-126-141-143-66 |  | 0.0051 |  | 0 |  | 0.0100 |  | 0.0093 |  | 0.0732 |
| H23 |  | 165-144-110-126-146-141-67 |  | 0.0051 |  | 0.0052 |  | 0 |  | 0 |  | 0 |
| H24 |  | 165-144-108-128-146-143-67 |  | 0 |  | 0.0052 |  | 0.0025 |  | 0 |  | 0 |
| H25 |  | 165-144-110-125-147-144-67 |  | 0 |  | 0.0207 |  | 0 |  | 0 |  | 0 |
| H26 |  | 165-144-110-127-147-141-67 |  | 0 |  | 0.0104 |  | 0.0025 |  | 0 |  | 0 |
| H27 |  | 165-144-110-125-146-145-67 |  | 0 |  | 0.0363 |  | 0.0075 |  | 0.0047 |  | 0.0244 |
| H28 |  | 166-144-109-126-146-143-67 |  | 0 |  | 0.0052 |  | 0 |  | 0 |  | 0 |
| H29 |  | 165-143-110-126-146-145-67 |  | 0 |  | 0.0104 |  | 0 |  | 0 |  | 0 |
| H30 |  | 165-144-109-125-147-143-67 |  | 0 |  | 0.0207 |  | 0 |  | 0 |  | 0 |
| H31 |  | 165-144-110-126-146-142-67 |  | 0 |  | 0.0155 |  | 0 |  | 0 |  | 0 |
| H32 |  | 165-144-110-127-146-144-67 |  | 0 |  | 0.0155 |  | 0 |  | 0.0093 |  | 0 |
| H33 |  | 165-144-111-126-148-143-67 |  | 0 |  | 0.0104 |  | 0.0075 |  | 0.0047 |  | 0 |
| H34 |  | 165-143-111-125-146-145-67 |  | 0 |  | 0.0052 |  | 0.0025 |  | 0.0047 |  | 0 |

Size variants from Pt87268-Pt36480-Pt26081-Pt15169-Pt71936-Pt30204-Pt1254.

**Table S2 (continued).** Chloroplast haplotype frequencies for *Pinus sylvestris* adults and offspring.

| Haplotype | | |  | Sample | | | | | | | | |
| --- | --- | --- | --- | --- | --- | --- | --- | --- | --- | --- | --- | --- |
|  |  |  |  | Adults | | |  | Seeds |  | Recruits | | |
| Code |  | Size variants |  | Native |  | Exotic |  |  |  | R1 |  | R2 |
| H35 |  | 164-144-110-125-146-144-67 |  | 0 |  | 0.0104 |  | 0 |  | 0 |  | 0 |
| H36 |  | 165-144-112-125-146-143-67 |  | 0 |  | 0.0052 |  | 0.0025 |  | 0.0047 |  | 0 |
| H37 |  | 165-144-109-126-147-143-67 |  | 0 |  | 0.0052 |  | 0 |  | 0 |  | 0 |
| H38 |  | 164-144-110-126-146-143-68 |  | 0 |  | 0.0052 |  | 0 |  | 0 |  | 0 |
| H39 |  | 168-144-110-123-146-145-67 |  | 0 |  | 0.0052 |  | 0 |  | 0 |  | 0 |
| H40 |  | 166-144-110-125-146-145-67 |  | 0 |  | 0.0104 |  | 0.0050 |  | 0.0093 |  | 0 |
| H41 |  | 165-144-110-127-146-142-68 |  | 0 |  | 0.0104 |  | 0.0025 |  | 0.0047 |  | 0 |
| H42 |  | 166-144-110-125-147-144-66 |  | 0 |  | 0.0052 |  | 0.0050 |  | 0 |  | 0 |
| H43 |  | 165-144-110-124-147-143-67 |  | 0 |  | 0.0052 |  | 0 |  | 0 |  | 0 |
| H44 |  | 166-144-110-125-148-144-66 |  | 0 |  | 0.0104 |  | 0 |  | 0 |  | 0 |
| H45 |  | 165-144-110-126-146-143-67 |  | 0 |  | 0.0466 |  | 0.0125 |  | 0.0047 |  | 0.0244 |
| H46 |  | 165-144-110-125-146-142-68 |  | 0 |  | 0.0155 |  | 0.0050 |  | 0 |  | 0 |
| H47 |  | 165-144-110-128-145-143-67 |  | 0 |  | 0.0052 |  | 0 |  | 0 |  | 0 |
| H48 |  | 164-144-109-125-145-142-67 |  | 0 |  | 0.0052 |  | 0 |  | 0 |  | 0 |
| H49 |  | 166-144-110-124-148-143-67 |  | 0 |  | 0.0052 |  | 0 |  | 0 |  | 0 |
| H50 |  | 167-144-110-125-146-144-67 |  | 0 |  | 0.0155 |  | 0 |  | 0 |  | 0 |
| H51 |  | 165-144-109-125-144-141-67 |  | 0 |  | 0.0052 |  | 0 |  | 0 |  | 0 |
| H52 |  | 165-144-110-127-146-142-66 |  | 0 |  | 0.0052 |  | 0 |  | 0 |  | 0 |
| H53 |  | 165-144-110-126-147-142-67 |  | 0 |  | 0.0104 |  | 0 |  | 0 |  | 0 |
| H54 |  | 165-144-110-127-147-143-67 |  | 0 |  | 0.0052 |  | 0 |  | 0 |  | 0 |
| H55 |  | 165-143-111-125-147-145-67 |  | 0 |  | 0.0052 |  | 0 |  | 0 |  | 0 |
| H56 |  | 167-144-110-125-146-147-67 |  | 0 |  | 0.0052 |  | 0 |  | 0 |  | 0 |
| H57 |  | 165-143-110-126-145-142-67 |  | 0 |  | 0.0052 |  | 0 |  | 0 |  | 0 |
| H58 |  | 165-144-109-126-146-141-67 |  | 0 |  | 0.0207 |  | 0.0075 |  | 0 |  | 0 |
| H59 |  | 165-143-110-125-147-144-66 |  | 0 |  | 0.0052 |  | 0 |  | 0 |  | 0 |
| H60 |  | 165-143-110-126-141-142-67 |  | 0 |  | 0.0052 |  | 0 |  | 0 |  | 0 |
| H61 |  | 165-144-110-127-146-142-67 |  | 0 |  | 0.0104 |  | 0.0025 |  | 0.0047 |  | 0 |
| H62 |  | 165-144-110-125-147-145-67 |  | 0 |  | 0.0155 |  | 0.0025 |  | 0 |  | 0 |
| H63 |  | 165-144-110-125-146-144-67 |  | 0 |  | 0.0104 |  | 0.0025 |  | 0 |  | 0 |
| H64 |  | 165-144-109-125-147-144-67 |  | 0 |  | 0.0104 |  | 0 |  | 0 |  | 0 |
| H65 |  | 166-144-110-125-146-143-66 |  | 0 |  | 0.0104 |  | 0.0025 |  | 0.0093 |  | 0 |
| H66 |  | 165-144-110-124-146-142-67 |  | 0 |  | 0.0052 |  | 0 |  | 0 |  | 0 |
| H67 |  | 165-143-110-126-141-144-67 |  | 0 |  | 0.0052 |  | 0 |  | 0 |  | 0 |

Size variants from Pt87268-Pt36480-Pt26081-Pt15169-Pt71936-Pt30204-Pt1254.

**Table S2 (continued).** Chloroplast haplotype frequencies for *Pinus sylvestris* adults and offspring.

| Haplotype | | |  | Sample | | | | | | | | |
| --- | --- | --- | --- | --- | --- | --- | --- | --- | --- | --- | --- | --- |
|  |  |  |  | Adults | | |  | Seeds |  | Recruits | | |
| Code |  | Size variants |  | Native |  | Exotic |  |  |  | R1 |  | R2 |
| H68 |  | 168-144-109-125-147-143-67 |  | 0 |  | 0.0052 |  | 0 |  | 0 |  | 0 |
| H69 |  | 165-144-110-127-145-145-67 |  | 0 |  | 0.0052 |  | 0 |  | 0 |  | 0 |
| H70 |  | 165-144-110-126-147-141-66 |  | 0 |  | 0.0052 |  | 0 |  | 0.0047 |  | 0 |
| H71 |  | 165-144-110-125-145-141-67 |  | 0 |  | 0.0104 |  | 0.0025 |  | 0 |  | 0 |
| H72 |  | 165-144-111-126-148-142-67 |  | 0 |  | 0.0052 |  | 0 |  | 0 |  | 0 |
| H73 |  | 166-144-110-124-146-143-66 |  | 0 |  | 0.0052 |  | 0 |  | 0 |  | 0 |
| H74 |  | 165-144-110-127-145-143-66 |  | 0 |  | 0.0052 |  | 0 |  | 0 |  | 0 |
| H75 |  | 165-144-110-125-146-141-66 |  | 0 |  | 0.0052 |  | 0 |  | 0.0047 |  | 0 |
| H76 |  | 166-144-110-124-144-144-67 |  | 0 |  | 0.0052 |  | 0 |  | 0 |  | 0 |
| H77 |  | 165-144-110-127-146-143-67 |  | 0 |  | 0.0104 |  | 0.0100 |  | 0 |  | 0 |
| H78 |  | 166-144-110-126-145-143-67 |  | 0 |  | 0.0052 |  | 0 |  | 0 |  | 0 |
| H79 |  | 166-144-110-124-147-143-67 |  | 0 |  | 0.0052 |  | 0 |  | 0 |  | 0 |
| H80 |  | 166-144-110-125-146-143-67 |  | 0 |  | 0.0052 |  | 0.0025 |  | 0 |  | 0 |
| H81 |  | 165-144-110-126-147-143-68 |  | 0 |  | 0.0052 |  | 0 |  | 0 |  | 0 |
| H82 |  | 165-143-110-125-145-142-67 |  | 0 |  | 0.0104 |  | 0 |  | 0 |  | 0 |
| H83 |  | 165-144-110-128-146-142-67 |  | 0 |  | 0.0052 |  | 0 |  | 0 |  | 0 |
| H84 |  | 165-144-110-126-147-143-66 |  | 0 |  | 0.0052 |  | 0 |  | 0 |  | 0 |
| H85 |  | 165-144-110-125-147-145-66 |  | 0 |  | 0.0052 |  | 0.0025 |  | 0.0093 |  | 0 |
| H86 |  | 165-144-110-127-147-142-66 |  | 0 |  | 0.0052 |  | 0 |  | 0 |  | 0 |
| H87 |  | 165-144-110-127-147-144-67 |  | 0 |  | 0.0052 |  | 0 |  | 0 |  | 0 |
| H88 |  | 165-144-110-126-146-144-67 |  | 0 |  | 0.0104 |  | 0.0025 |  | 0 |  | 0.0244 |
| H89 |  | 165-144-110-126-147-145-67 |  | 0 |  | 0.0052 |  | 0 |  | 0 |  | 0 |
| H90 |  | 166-144-110-125-146-147-67 |  | 0 |  | 0.0052 |  | 0 |  | 0 |  | 0 |
| H91 |  | 165-144-110-126-147-144-66 |  | 0 |  | 0.0052 |  | 0 |  | 0 |  | 0 |
| H92 |  | 164-144-110-126-146-144-67 |  | 0 |  | 0.0052 |  | 0 |  | 0 |  | 0 |
| H93 |  | 168-144-110-125-147-143-66 |  | 0 |  | 0.0052 |  | 0 |  | 0 |  | 0 |
| H94 |  | 167-144-109-128-145-143-67 |  | 0 |  | 0.0052 |  | 0 |  | 0 |  | 0 |
| H95 |  | 165-144-109-128-146-142-66 |  | 0 |  | 0.0104 |  | 0 |  | 0 |  | 0 |
| H96 |  | 166-144-110-126-146-143-67 |  | 0 |  | 0.0104 |  | 0.0025 |  | 0 |  | 0 |
| H97 |  | 165-144-109-126-146-143-67 |  | 0 |  | 0.0104 |  | 0 |  | 0 |  | 0 |
| H98 |  | 165-152-111-127-145-144-67 |  | 0 |  | 0.0052 |  | 0 |  | 0 |  | 0 |
| H99 |  | 165-144-109-126-147-141-67 |  | 0 |  | 0.0052 |  | 0 |  | 0 |  | 0 |
| H100 |  | 166-144-110-124-147-145-66 |  | 0 |  | 0.0052 |  | 0 |  | 0.0047 |  | 0 |

Size variants from Pt87268-Pt36480-Pt26081-Pt15169-Pt71936-Pt30204-Pt1254.

**Table S2 (continued).** Chloroplast haplotype frequencies for *Pinus sylvestris* adults and offspring.

| Haplotype | | |  | Sample | | | | | | | | |
| --- | --- | --- | --- | --- | --- | --- | --- | --- | --- | --- | --- | --- |
|  |  |  |  | Adults | | |  | Seeds |  | Recruits | | |
| Code |  | Size variants |  | Native |  | Exotic |  |  |  | R1 |  | R2 |
| H101 |  | 166-144-110-125-146-144-67 |  | 0 |  | 0.0207 |  | 0.0125 |  | 0.0093 |  | 0 |
| H102 |  | 165-144-111-126-146-143-68 |  | 0 |  | 0.0052 |  | 0 |  | 0 |  | 0 |
| H103 |  | 165-144-109-127-146-143-67 |  | 0 |  | 0.0052 |  | 0 |  | 0 |  | 0 |
| H104 |  | 165-144-110-125-148-145-67 |  | 0 |  | 0.0052 |  | 0 |  | 0 |  | 0 |
| H105 |  | 166-144-110-127-146-142-67 |  | 0 |  | 0.0052 |  | 0 |  | 0 |  | 0 |
| H106 |  | 166-143-110-125-146-144-68 |  | 0 |  | 0.0052 |  | 0.0025 |  | 0 |  | 0 |
| H107 |  | 165-152-111-127-145-145-67 |  | 0 |  | 0.0052 |  | 0 |  | 0 |  | 0 |
| H108 |  | 165-144-110-125-148-144-67 |  | 0 |  | 0.0052 |  | 0.0025 |  | 0 |  | 0 |
| H109 |  | 165-144-110-128-146-146-67 |  | 0 |  | 0.0052 |  | 0 |  | 0 |  | 0 |
| H110 |  | 165-144-110-125-146-142-67 |  | 0 |  | 0.0052 |  | 0 |  | 0 |  | 0 |
| H111 |  | 165-143-110-126-141-143-67 |  | 0 |  | 0.0052 |  | 0.0025 |  | 0.0047 |  | 0 |
| H112 |  | 165-144-110-126-147-143-67 |  | 0 |  | 0.0155 |  | 0.0075 |  | 0.0140 |  | 0 |
| H113 |  | 165-144-110-125-146-143-67 |  | 0 |  | 0.0052 |  | 0.0025 |  | 0 |  | 0 |
| H114 |  | 166-144-110-125-146-146-67 |  | 0 |  | 0.0052 |  | 0 |  | 0 |  | 0 |
| H115 |  | 165-144-109-125-146-142-67 |  | 0 |  | 0.0052 |  | 0 |  | 0 |  | 0 |
| H116 |  | 165-144-110-126-145-143-68 |  | 0 |  | 0.0052 |  | 0 |  | 0 |  | 0 |
| H117 |  | 166-144-109-125-146-145-67 |  | 0 |  | 0.0052 |  | 0 |  | 0 |  | 0 |
| H118 |  | 166-144-110-124-147-145-67 |  | 0 |  | 0.0052 |  | 0 |  | 0 |  | 0 |
| H119 |  | 165-144-110-125-147-144-66 |  | 0 |  | 0.0052 |  | 0 |  | 0 |  | 0 |
| H120 |  | 165-143-111-126-145-144-66 |  | 0 |  | 0.0104 |  | 0 |  | 0 |  | 0 |
| H121 |  | 166-144-110-124-146-143-67 |  | 0 |  | 0.0052 |  | 0 |  | 0 |  | 0 |
| H122 |  | 168-144-110-126-148-143-67 |  | 0 |  | 0.0104 |  | 0 |  | 0 |  | 0 |
| H123 |  | 168-144-111-126-147-145-67 |  | 0 |  | 0.0052 |  | 0 |  | 0.0047 |  | 0 |
| H124 |  | 166-144-110-127-144-144-68 |  | 0 |  | 0.0052 |  | 0 |  | 0 |  | 0 |
| H125 |  | 165-144-109-126-147-142-67 |  | 0 |  | 0.0052 |  | 0.0025 |  | 0 |  | 0 |
| H126 |  | 166-144-110-128-147-145-67 |  | 0 |  | 0.0052 |  | 0 |  | 0 |  | 0 |
| H127 |  | 165-143-110-125-144-144-67 |  | 0 |  | 0.0052 |  | 0 |  | 0 |  | 0 |
| H128 |  | 165-144-109-125-145-143-67 |  | 0 |  | 0.0052 |  | 0 |  | 0 |  | 0 |
| H129 |  | 164-144-110-127-146-144-67 |  | 0 |  | 0.0052 |  | 0 |  | 0 |  | 0 |
| H130 |  | 165-144-110-124-146-144-67 |  | 0 |  | 0.0052 |  | 0 |  | 0 |  | 0 |
| H131 |  | 167-144-110-125-146-142-68 |  | 0 |  | 0.0052 |  | 0 |  | 0 |  | 0 |
| H132 |  | 164-144-110-126-146-143-67 |  | 0 |  | 0.0052 |  | 0 |  | 0 |  | 0 |
| H133 |  | 165-143-110-125-147-143-68 |  | 0 |  | 0.0052 |  | 0 |  | 0 |  | 0 |

Size variants from Pt87268-Pt36480-Pt26081-Pt15169-Pt71936-Pt30204-Pt1254.

**Table S2 (continued).** Chloroplast haplotype frequencies for *Pinus sylvestris* adults and offspring.

| Haplotype | | |  | Sample | | | | | | | | |
| --- | --- | --- | --- | --- | --- | --- | --- | --- | --- | --- | --- | --- |
|  |  |  |  | Adults | | |  | Seeds |  | Recruits | | |
| Code |  | Size variants |  | Native |  | Exotic |  |  |  | R1 |  | R2 |
| H134 |  | 166-144-109-127-146-143-67 |  | 0 |  | 0 |  | 0.0025 |  | 0 |  | 0 |
| H135 |  | 166-144-110-127-146-143-67 |  | 0 |  | 0 |  | 0.0025 |  | 0 |  | 0 |
| H136 |  | 165-144-109-127-146-144-67 |  | 0 |  | 0 |  | 0.0025 |  | 0 |  | 0 |
| H137 |  | 166-144-110-125-146-144-68 |  | 0 |  | 0 |  | 0.0075 |  | 0 |  | 0 |
| H138 |  | 166-144-110-126-146-145-67 |  | 0 |  | 0 |  | 0.0025 |  | 0.0047 |  | 0 |
| H139 |  | 166-144-110-123-144-144-67 |  | 0 |  | 0 |  | 0.0025 |  | 0 |  | 0 |
| H140 |  | 165-144-110-125-147-146-67 |  | 0 |  | 0 |  | 0.0025 |  | 0.0093 |  | 0 |
| H141 |  | 166-144-110-124-144-143-67 |  | 0 |  | 0 |  | 0.0025 |  | 0 |  | 0 |
| H142 |  | 165-144-110-127-147-145-66 |  | 0 |  | 0 |  | 0.0025 |  | 0 |  | 0 |
| H143 |  | 165-144-109-127-145-142-67 |  | 0 |  | 0 |  | 0.0050 |  | 0 |  | 0 |
| H144 |  | 165-144-110-125-146-143-68 |  | 0 |  | 0 |  | 0.0025 |  | 0 |  | 0 |
| H145 |  | 165-144-110-127-147-142-67 |  | 0 |  | 0 |  | 0.0050 |  | 0.0140 |  | 0.0244 |
| H146 |  | 165-143-110-126-146-146-67 |  | 0 |  | 0 |  | 0.0025 |  | 0 |  | 0 |
| H147 |  | 166-144-110-125-147-145-67 |  | 0 |  | 0 |  | 0.0050 |  | 0 |  | 0 |
| H148 |  | 165-144-110-125-145-143-68 |  | 0 |  | 0 |  | 0.0025 |  | 0 |  | 0 |
| H149 |  | 166-144-110-126-147-143-67 |  | 0 |  | 0 |  | 0.0025 |  | 0 |  | 0 |
| H150 |  | 165-143-110-126-146-144-68 |  | 0 |  | 0 |  | 0.0025 |  | 0 |  | 0 |
| H151 |  | 167-144-110-125-146-146-67 |  | 0 |  | 0 |  | 0.0025 |  | 0 |  | 0 |
| H152 |  | 165-144-110-126-147-144-67 |  | 0 |  | 0 |  | 0.0025 |  | 0 |  | 0 |
| H153 |  | 165-144-109-124-147-142-66 |  | 0 |  | 0 |  | 0.0025 |  | 0 |  | 0 |
| H154 |  | 165-144-110-124-148-145-67 |  | 0 |  | 0 |  | 0.0025 |  | 0 |  | 0 |
| H155 |  | 165-144-110-124-144-144-70 |  | 0 |  | 0 |  | 0.0050 |  | 0 |  | 0 |
| H156 |  | 165-144-110-126-146-146-67 |  | 0 |  | 0 |  | 0.0025 |  | 0 |  | 0 |
| H157 |  | 167-144-110-125-146-141-66 |  | 0 |  | 0 |  | 0.0025 |  | 0 |  | 0 |
| H158 |  | 165-144-110-125-145-144-67 |  | 0 |  | 0 |  | 0.0050 |  | 0 |  | 0 |
| H159 |  | 166-144-110-126-145-144-67 |  | 0 |  | 0 |  | 0.0025 |  | 0 |  | 0 |
| H160 |  | 167-144-110-125-146-143-67 |  | 0 |  | 0 |  | 0 |  | 0.0093 |  | 0.0244 |
| H161 |  | 165-144-109-125-148-145-67 |  | 0 |  | 0 |  | 0 |  | 0.0093 |  | 0 |
| H162 |  | 167-145-108-117-147-143-67 |  | 0 |  | 0 |  | 0 |  | 0.0047 |  | 0 |
| H163 |  | 167-145-108-117-147-146-67 |  | 0 |  | 0 |  | 0 |  | 0.0047 |  | 0 |
| H164 |  | 167-145-108-117-147-142-66 |  | 0 |  | 0 |  | 0 |  | 0.0047 |  | 0 |
| H165 |  | 165-144-110-125-147-146-66 |  | 0 |  | 0 |  | 0 |  | 0.0047 |  | 0 |
| H166 |  | 168-145-108-117-147-143-66 |  | 0 |  | 0 |  | 0 |  | 0.0047 |  | 0 |

Size variants from Pt87268-Pt36480-Pt26081-Pt15169-Pt71936-Pt30204-Pt1254.

**Table S2 (continued).** Chloroplast haplotype frequencies for *Pinus sylvestris* adults and offspring.

| Haplotype | | |  | Sample | | | | | | | | |
| --- | --- | --- | --- | --- | --- | --- | --- | --- | --- | --- | --- | --- |
|  |  |  |  | Adults | | |  | Seeds |  | Recruits | | |
| Code |  | Size variants |  | Native |  | Exotic |  |  |  | R1 |  | R2 |
| H167 |  | 168-145-108-117-146-143-67 |  | 0 |  | 0 |  | 0 |  | 0.0047 |  | 0.0244 |
| H168 |  | 168-145-108-117-146-145-67 |  | 0 |  | 0 |  | 0 |  | 0.0047 |  | 0 |
| H169 |  | 165-144-110-126-146-142-66 |  | 0 |  | 0 |  | 0 |  | 0.0047 |  | 0 |
| H170 |  | 165-144-110-125-145-145-67 |  | 0 |  | 0 |  | 0 |  | 0.0047 |  | 0 |
| H171 |  | 165-143-111-125-146-144-67 |  | 0 |  | 0 |  | 0 |  | 0.0047 |  | 0 |
| H172 |  | 165-144-110-126-145-144-67 |  | 0 |  | 0 |  | 0 |  | 0.0047 |  | 0 |
| H173 |  | 165-144-110-126-145-141-67 |  | 0 |  | 0 |  | 0 |  | 0.0047 |  | 0 |
| H174 |  | 166-144-110-127-145-143-67 |  | 0 |  | 0 |  | 0 |  | 0.0047 |  | 0 |
| H175 |  | 166-144-110-127-147-142-67 |  | 0 |  | 0 |  | 0 |  | 0.0047 |  | 0 |
| H176 |  | 166-144-110-128-144-143-67 |  | 0 |  | 0 |  | 0 |  | 0.0047 |  | 0 |
| H177 |  | 166-144-110-127-144-144-67 |  | 0 |  | 0 |  | 0 |  | 0 |  | 0.0244 |
| H178 |  | 166-144-110-124-147-144-66 |  | 0 |  | 0 |  | 0 |  | 0 |  | 0.0244 |
| H179 |  | 166-144-110-126-147-145-66 |  | 0 |  | 0 |  | 0 |  | 0 |  | 0.0244 |
| H180 |  | 168-144-110-124-147-142-67 |  | 0 |  | 0 |  | 0 |  | 0 |  | 0.0244 |

Size variants from Pt87268-Pt36480-Pt26081-Pt15169-Pt71936-Pt30204-Pt1254.

**Table S3.** Allelic frequencies for nSSR loci in *Pinus pinaster*.

| nSSR | | |  | Sample | | | | | | | | | | |
| --- | --- | --- | --- | --- | --- | --- | --- | --- | --- | --- | --- | --- | --- | --- |
| Locus |  | Allele |  | Adults | | |  | Seeds |  | Recruits | | | | |
|  |  |  |  | Native |  | Exotic |  |  |  | R1 |  | R2 |  | R3 |
| NZPR544 |  | 243 |  | 0 |  | *0.0079* |  | 0 |  | 0 |  | 0 |  | 0 |
|  |  | 245 |  | 0.5000 |  | 0.3268 |  | 0.5339 |  | 0.5213 |  | 0.4227 |  | 0.3750 |
|  |  | 247 |  | 0 |  | *0.0118* |  | 0 |  | 0 |  | 0 |  | 0 |
|  |  | 249 |  | 0.5000 |  | 0.6417 |  | 0.4654 |  | 0.4787 |  | 0.5773 |  | 0.6250 |
|  |  | 251 |  | 0 |  | *0.0039* |  | 0.0008 |  | 0 |  | 0 |  | 0 |
|  |  | 271 |  | 0 |  | *0.0079* |  | 0 |  | 0 |  | 0 |  | 0 |
|  |  |  |  |  |  |  |  |  |  |  |  |  |  |  |
| SsrPt_ctg275 |  | 112 |  | 0 |  | *0.0882* |  | 0 |  | 0 |  | 0 |  | 0 |
|  |  | 114 |  | 0.0211 |  | 0.2647 |  | 0.0165 |  | 0.0106 |  | 0.0521 |  | 0.0116 |
|  |  | 118 |  | 0.6895 |  | 0.3713 |  | 0.7291 |  | 0.7660 |  | 0.7240 |  | 0.7558 |
|  |  | 120 |  | 0.0316 |  | 0.0552 |  | 0.0047 |  | 0.0053 |  | 0 |  | 0.0116 |
|  |  | 122 |  | 0 |  | *0.0074* |  | 0.0032 |  | 0 |  | 0 |  | 0 |
|  |  | 124 |  | 0 |  | 0 |  | 0.0016 |  | 0 |  | 0 |  | 0 |
|  |  | 126 |  | 0 |  | *0.0074* |  | 0 |  | 0 |  | 0 |  | 0 |
|  |  | 128 |  | 0.0158 |  | 0.0294 |  | 0.0205 |  | 0 |  | 0 |  | 0 |
|  |  | 130 |  | 0.0263 |  | 0.0515 |  | 0.0039 |  | 0.0053 |  | 0.0052 |  | 0.0116 |
|  |  | 132 |  | 0.0211 |  | 0.0735 |  | 0.0449 |  | 0.0160 |  | 0.0573 |  | 0.0116 |
|  |  | 134 |  | 0.1632 |  | 0.0294 |  | 0.1488 |  | 0.1649 |  | 0.1458 |  | 0.1744 |
|  |  | 136 |  | 0.0316 |  | 0.0221 |  | 0.0268 |  | 0.0319 |  | 0.0156 |  | 0.0233 |
|  |  |  |  |  |  |  |  |  |  |  |  |  |  |  |
| A6F03 |  | 234 |  | 0 |  | *0.0187* |  | 0.0047 |  | 0.0054 |  | 0.0104 |  | 0.0341 |
|  |  | 240 |  | 0.1053 |  | 0.0784 |  | 0.0529 |  | 0.0591 |  | 0.0156 |  | 0.0455 |
|  |  | 244 |  | 0.0474 |  | 0.4478 |  | 0.1082 |  | 0.0538 |  | 0.0469 |  | 0.0114 |
|  |  | 246 |  | 0.0053 |  | 0.1269 |  | 0.0024 |  | 0.0054 |  | 0 |  | 0 |
|  |  | 248 |  | 0.0421 |  | 0.1866 |  | 0.0332 |  | 0.0108 |  | 0.0625 |  | 0.0455 |
|  |  | 250 |  | 0.0684 |  | 0.0261 |  | 0.0269 |  | 0.0108 |  | 0.0156 |  | 0.0114 |
|  |  | 252 |  | 0.7316 |  | 0.1119 |  | 0.7717 |  | 0.8495 |  | 0.8490 |  | 0.8523 |
|  |  | 254 |  | 0 |  | *0.0037* |  | 0 |  | 0.0054 |  | 0 |  | 0 |
|  |  |  |  |  |  |  |  |  |  |  |  |  |  |  |
| RPtest11 |  | 205 |  | 0.3710 |  | 0.2500 |  | 0.4683 |  | 0.4894 |  | 0.3281 |  | 0.3023 |
|  |  | 208 |  | 0.5215 |  | 0.3493 |  | 0.4691 |  | 0.3883 |  | 0.5729 |  | 0.6512 |
|  |  | 211 |  | 0.0860 |  | 0.2243 |  | 0.0563 |  | 0.1170 |  | 0.0990 |  | 0.0465 |
|  |  | 214 |  | 0.0215 |  | 0.1765 |  | 0.0063 |  | 0.0053 |  | 0 |  | 0 |

Frequencies of alleles specific to the native or exotic adult samples are highlighted in italics.

**Table S3 (continued).** Allelic frequencies for nSSR loci in *Pinus pinaster*.

| nSSR | | |  | Sample | | | | | | | | | | |
| --- | --- | --- | --- | --- | --- | --- | --- | --- | --- | --- | --- | --- | --- | --- |
| Locus |  | Allele |  | Adults | | |  | Seeds |  | Recruits | | | | |
|  |  |  |  | Native |  | Exotic |  |  |  | R1 |  | R2 |  | R3 |
| SsrPt_ctg4363 |  | 87 |  | 0 |  | *0.0221* |  | 0.0008 |  | 0.0068 |  | 0 |  | 0 |
|  |  | 89 |  | 0 |  | *0.0147* |  | 0 |  | 0 |  | 0 |  | 0 |
|  |  | 91 |  | 0 |  | *0.0074* |  | 0 |  | 0 |  | 0 |  | 0 |
|  |  | 93 |  | 0.1212 |  | 0.2721 |  | 0.1321 |  | 0.0405 |  | 0 |  | 0.0341 |
|  |  | 95 |  | 0.4444 |  | 0.2574 |  | 0.3895 |  | 0.3851 |  | 0.4624 |  | 0.3636 |
|  |  | 97 |  | 0 |  | *0.0699* |  | 0.0083 |  | 0.0068 |  | 0.0054 |  | 0 |
|  |  | 99 |  | 0.2323 |  | 0.2500 |  | 0.2799 |  | 0.3987 |  | 0.4409 |  | 0.4091 |
|  |  | 101 |  | 0.1970 |  | 0.0147 |  | 0.1885 |  | 0.1622 |  | 0.0914 |  | 0.1932 |
|  |  | 103 |  | 0.0051 |  | 0.0809 |  | 0.0008 |  | 0 |  | 0 |  | 0 |
|  |  | 105 |  | 0 |  | *0.0074* |  | 0 |  | 0 |  | 0 |  | 0 |
|  |  | 109 |  | 0 |  | *0.0037* |  | 0 |  | 0 |  | 0 |  | 0 |
|  |  |  |  |  |  |  |  |  |  |  |  |  |  |  |
| NZPR1078 |  | 328 |  | 0.0050 |  | 0.1008 |  | 0.0016 |  | 0 |  | 0 |  | 0 |
|  |  | 330 |  | 0.2921 |  | 0.1279 |  | 0.3118 |  | 0.3387 |  | 0.2850 |  | 0.2841 |
|  |  | 332 |  | 0.4703 |  | 0.5504 |  | 0.3789 |  | 0.3710 |  | 0.5376 |  | 0.4205 |
|  |  | 334 |  | 0 |  | 0 |  | 0.0016 |  | 0 |  | 0 |  | 0 |
|  |  | 338 |  | 0 |  | 0 |  | 0.0008 |  | 0 |  | 0 |  | 0 |
|  |  | 340 |  | 0.2327 |  | 0.2209 |  | 0.2995 |  | 0.2903 |  | 0.1774 |  | 0.2955 |
|  |  | 342 |  | 0 |  | 0 |  | 0.0057 |  | 0 |  | 0 |  | 0 |
|  |  |  |  |  |  |  |  |  |  |  |  |  |  |  |
| epi3 |  | 219 |  | 0.0099 |  | 0.1157 |  | 0.0090 |  | 0.0163 |  | 0.0161 |  | 0.0227 |
|  |  | 221 |  | 0.1089 |  | 0.1828 |  | 0.1042 |  | 0.1685 |  | 0.1720 |  | 0.1591 |
|  |  | 223 |  | 0 |  | *0.0261* |  | 0 |  | 0 |  | 0 |  | 0 |
|  |  | 227 |  | 0.0743 |  | 0.0522 |  | 0.0195 |  | 0.0272 |  | 0.0269 |  | 0 |
|  |  | 229 |  | 0.2030 |  | 0.2836 |  | 0.2239 |  | 0.1467 |  | 0.2043 |  | 0.1932 |
|  |  | 231 |  | 0.1238 |  | 0.2052 |  | 0.0920 |  | 0.0707 |  | 0.0914 |  | 0.1477 |
|  |  | 233 |  | 0.3119 |  | 0.0933 |  | 0.3722 |  | 0.4130 |  | 0.3602 |  | 0.3864 |
|  |  | 235 |  | 0.0545 |  | 0.0149 |  | 0.0513 |  | 0.0054 |  | 0.0323 |  | 0.0227 |
|  |  | 237 |  | 0.1139 |  | 0.0224 |  | 0.1279 |  | 0.1522 |  | 0.0914 |  | 0.0682 |
|  |  | 241 |  | 0 |  | *0.0037* |  | 0 |  | 0 |  | 0 |  | 0 |
|  |  | 243 |  | 0 |  | 0 |  | 0 |  | 0 |  | 0.0054 |  | 0 |

Frequencies of alleles specific to the native or exotic adult samples are highlighted in italics.

**Table S3 (continued).** Allelic frequencies for nSSR loci in *Pinus pinaster*.

| nSSR | | |  | Sample | | | | | | | | | | |
| --- | --- | --- | --- | --- | --- | --- | --- | --- | --- | --- | --- | --- | --- | --- |
| Locus |  | Allele |  | Adults | | |  | Seeds |  | Recruits | | | | |
|  |  |  |  | Native |  | Exotic |  |  |  | R1 |  | R2 |  | R3 |
| FRPP94 |  | 131 |  | 0.4604 |  | 0.1993 |  | 0.4625 |  | 0.5000 |  | 0.5591 |  | 0.5796 |
|  |  | 135 |  | 0 |  | 0 |  | 0.0008 |  | 0 |  | 0 |  | 0 |
|  |  | 137 |  | 0.1386 |  | 0.1993 |  | 0.1018 |  | 0.1278 |  | 0.1505 |  | 0.1250 |
|  |  | 141 |  | 0.0149 |  | 0.0639 |  | 0.0081 |  | 0 |  | 0 |  | 0 |
|  |  | 145 |  | 0 |  | *0.0038* |  | 0.0008 |  | 0 |  | 0 |  | 0 |
|  |  | 147 |  | 0.3267 |  | 0.3045 |  | 0.4064 |  | 0.3333 |  | 0.2581 |  | 0.2614 |
|  |  | 149 |  | 0.0099 |  | 0.0451 |  | 0.0016 |  | 0.0056 |  | 0 |  | 0 |
|  |  | 151 |  | 0.0149 |  | 0.0038 |  | 0.0024 |  | 0 |  | 0 |  | 0 |
|  |  | 153 |  | 0 |  | *0.0940* |  | 0.0033 |  | 0 |  | 0 |  | 0 |
|  |  | 155 |  | 0 |  | *0.0150* |  | 0 |  | 0 |  | 0 |  | 0 |
|  |  | 157 |  | 0.0297 |  | 0.0451 |  | 0.0114 |  | 0.0333 |  | 0.0269 |  | 0.0341 |
|  |  | 159 |  | 0.0050 |  | 0.0263 |  | 0.0008 |  | 0 |  | 0.0054 |  | 0 |
|  |  |  |  |  |  |  |  |  |  |  |  |  |  |  |
| NZPR413 |  | 161 |  | *0.0103* |  | 0 |  | 0.0166 |  | 0.0053 |  | 0 |  | 0 |
|  |  | 163 |  | 0.1289 |  | 0.3024 |  | 0.1507 |  | 0.1895 |  | 0.1505 |  | 0.0595 |
|  |  | 169 |  | 0.8608 |  | 0.6815 |  | 0.8328 |  | 0.8053 |  | 0.8495 |  | 0.9405 |
|  |  | 171 |  | 0 |  | *0.0040* |  | 0 |  | 0 |  | 0 |  | 0 |
|  |  | 187 |  | 0 |  | *0.0121* |  | 0 |  | 0 |  | 0 |  | 0 |
|  |  |  |  |  |  |  |  |  |  |  |  |  |  |  |
| gPp14 |  | 188 |  | 0.0206 |  | 0.0195 |  | 0.0598 |  | 0.0474 |  | 0.0161 |  | 0.0357 |
|  |  | 191 |  | 0 |  | *0.0039* |  | 0.0033 |  | 0 |  | 0 |  | 0 |
|  |  | 197 |  | 0.0464 |  | 0.0742 |  | 0.0382 |  | 0.0263 |  | 0.0215 |  | 0.0476 |
|  |  | 200 |  | 0.7165 |  | 0.7422 |  | 0.6761 |  | 0.7000 |  | 0.7688 |  | 0.8452 |
|  |  | 209 |  | 0.2165 |  | 0.1602 |  | 0.2226 |  | 0.2263 |  | 0.1936 |  | 0.0714 |

Frequencies of alleles specific to the native or exotic adult samples are highlighted in italics.

**Table S3 (continued).** Allelic frequencies for nSSR loci in *Pinus pinaster*.

| nSSR | | |  | Sample | | | | | | | | | | |
| --- | --- | --- | --- | --- | --- | --- | --- | --- | --- | --- | --- | --- | --- | --- |
| Locus |  | Allele |  | Adults | | |  | Seeds |  | Recruits | | | | |
|  |  |  |  | Native |  | Exotic |  |  |  | R1 |  | R2 |  | R3 |
| pEST2669 |  | 141 |  | 0.2062 |  | 0.0892 |  | 0.1639 |  | 0.1000 |  | 0.1129 |  | 0.1191 |
|  |  | 143 |  | 0.2629 |  | 0.2830 |  | 0.2351 |  | 0.1632 |  | 0.4086 |  | 0.2619 |
|  |  | 145 |  | 0.4742 |  | 0.5543 |  | 0.5935 |  | 0.7263 |  | 0.4677 |  | 0.5714 |
|  |  | 147 |  | 0.0052 |  | 0.0078 |  | 0.0033 |  | 0.0053 |  | 0 |  | 0 |
|  |  | 151 |  | *0.0464* |  | 0 |  | 0 |  | 0 |  | 0 |  | 0 |
|  |  | 153 |  | 0 |  | *0.0039* |  | 0 |  | 0 |  | 0 |  | 0 |
|  |  | 155 |  | 0 |  | *0.0039* |  | 0.0008 |  | 0 |  | 0 |  | 0 |
|  |  | 157 |  | 0 |  | *0.0155* |  | 0.0025 |  | 0.0053 |  | 0.0108 |  | 0.0476 |
|  |  | 159 |  | 0.0052 |  | 0.0039 |  | 0 |  | 0 |  | 0 |  | 0 |
|  |  | 163 |  | 0 |  | *0.0039* |  | 0 |  | 0 |  | 0 |  | 0 |
|  |  | 165 |  | 0 |  | *0.0349* |  | 0.0008 |  | 0 |  | 0 |  | 0 |
|  |  |  |  |  |  |  |  |  |  |  |  |  |  |  |
| epi5 |  | 185 |  | 0.0670 |  | 0.1953 |  | 0.0290 |  | 0.0579 |  | 0.0161 |  | 0.0119 |
|  |  | 191 |  | 0 |  | *0.0781* |  | 0 |  | 0.0053 |  | 0 |  | 0.0119 |
|  |  | 193 |  | 0 |  | *0.0195* |  | 0 |  | 0 |  | 0 |  | 0 |
|  |  | 197 |  | 0 |  | *0.0156* |  | 0.0008 |  | 0 |  | 0 |  | 0 |
|  |  | 199 |  | 0.7732 |  | 0.6719 |  | 0.7546 |  | 0.7579 |  | 0.9032 |  | 0.8929 |
|  |  | 201 |  | 0 |  | *0.0039* |  | 0 |  | 0 |  | 0 |  | 0 |
|  |  | 203 |  | 0.1598 |  | 0.0156 |  | 0.2156 |  | 0.1790 |  | 0.0807 |  | 0.0833 |

Frequencies of alleles specific to the native or exotic adult samples are highlighted in italics.

**Table S4.** Allelic frequencies for nSSR loci in *Pinus sylvestris*.

| nSSR | | |  | Sample | | | | | | | | |
| --- | --- | --- | --- | --- | --- | --- | --- | --- | --- | --- | --- | --- |
| Locus |  | Allele |  | Adults | | |  | Seeds |  | Recruits | | |
|  |  |  |  | Native |  | Exotic |  |  |  | R1 |  | R2 |
| psyl17 |  | 212 |  | 0 |  | *0.0105* |  | 0.0013 |  | 0 |  | 0 |
|  |  | 214 |  | 0 |  | *0.0026* |  | 0 |  | 0 |  | 0 |
|  |  | 218 |  | 0 |  | *0.0105* |  | 0.0065 |  | 0.0024 |  | 0 |
|  |  | 220 |  | 0.0128 |  | 0.0445 |  | 0.0196 |  | 0.0308 |  | 0.0250 |
|  |  | 222 |  | 0.1811 |  | 0.1440 |  | 0.1361 |  | 0.1374 |  | 0.2000 |
|  |  | 224 |  | 0.1735 |  | 0.2225 |  | 0.1976 |  | 0.1611 |  | 0.2250 |
|  |  | 226 |  | 0.3954 |  | 0.2094 |  | 0.2984 |  | 0.3460 |  | 0.2500 |
|  |  | 228 |  | 0.2372 |  | 0.2618 |  | 0.3298 |  | 0.3057 |  | 0.2875 |
|  |  | 230 |  | 0 |  | *0.0576* |  | 0.0039 |  | 0.0047 |  | 0.0125 |
|  |  | 232 |  | 0 |  | *0.0262* |  | 0.0039 |  | 0.0024 |  | 0 |
|  |  | 234 |  | 0 |  | *0.0079* |  | 0.0026 |  | 0 |  | 0 |
|  |  | 236 |  | 0 |  | *0.0026* |  | 0 |  | 0 |  | 0 |
|  |  | 238 |  | 0 |  | 0 |  | 0 |  | 0.0047 |  | 0 |
|  |  | 240 |  | 0 |  | 0 |  | 0 |  | 0.0047 |  | 0 |
|  |  |  |  |  |  |  |  |  |  |  |  |  |
| psyl18 |  | 288 |  | 0 |  | *0.0026* |  | 0 |  | 0 |  | 0 |
|  |  | 294 |  | 0.1352 |  | 0.0158 |  | 0.0840 |  | 0.0755 |  | 0.1000 |
|  |  | 297 |  | 0.8316 |  | 0.9579 |  | 0.9029 |  | 0.8915 |  | 0.8750 |
|  |  | 300 |  | 0.0332 |  | 0.0079 |  | 0.0092 |  | 0.0212 |  | 0.0250 |
|  |  | 303 |  | 0 |  | *0.0158* |  | 0.0039 |  | 0.0024 |  | 0 |
|  |  | 306 |  | 0 |  | 0 |  | 0 |  | 0.0094 |  | 0 |
|  |  |  |  |  |  |  |  |  |  |  |  |  |
| psyl25 |  | 214 |  | 0 |  | *0.0026* |  | 0 |  | 0 |  | 0 |
|  |  | 217 |  | 1.0000 |  | 0.9974 |  | 1.0000 |  | 1.0000 |  | 1.0000 |
|  |  |  |  |  |  |  |  |  |  |  |  |  |
| psyl42 |  | 169 |  | 0.4362 |  | 0.2884 |  | 0.4411 |  | 0.4115 |  | 0.4250 |
|  |  | 173 |  | 0 |  | *0.0212* |  | 0.0013 |  | 0.0048 |  | 0.0125 |
|  |  | 175 |  | 0.0995 |  | 0.3783 |  | 0.0851 |  | 0.0861 |  | 0.0375 |
|  |  | 177 |  | 0.4643 |  | 0.3042 |  | 0.4725 |  | 0.4928 |  | 0.5250 |
|  |  | 179 |  | 0 |  | *0.0027* |  | 0 |  | 0.0024 |  | 0 |
|  |  | 181 |  | 0 |  | *0.0053* |  | 0 |  | 0.0024 |  | 0 |

Frequencies of alleles specific to the native or exotic adult samples are highlighted in italics.

**Table S4 (continued).** Allelic frequencies for nSSR loci in *Pinus sylvestris*.

| nSSR | | |  | Sample | | | | | | | | |
| --- | --- | --- | --- | --- | --- | --- | --- | --- | --- | --- | --- | --- |
| Locus |  | Allele |  | Adults | | |  | Seeds |  | Recruits | | |
|  |  |  |  | Native |  | Exotic |  |  |  | R1 |  | R2 |
| psyl57 |  | 189 |  | 0.0513 |  | 0.0707 |  | 0.0183 |  | 0.0616 |  | 0.0625 |
|  |  | 192 |  | 0 |  | *0.0236* |  | 0.0013 |  | 0.0047 |  | 0.0125 |
|  |  | 195 |  | 0.0282 |  | 0.1361 |  | 0.0261 |  | 0.0308 |  | 0.0375 |
|  |  | 198 |  | 0.3923 |  | 0.5576 |  | 0.4791 |  | 0.4313 |  | 0.4500 |
|  |  | 201 |  | 0.5282 |  | 0.1885 |  | 0.4752 |  | 0.4692 |  | 0.4375 |
|  |  | 204 |  | 0 |  | *0.0209* |  | 0 |  | 0.0024 |  | 0 |
|  |  | 207 |  | 0 |  | *0.0026* |  | 0 |  | 0 |  | 0 |
|  |  |  |  |  |  |  |  |  |  |  |  |  |
| psyl36 |  | 249 |  | 0.0100 |  | 0.0622 |  | 0.0463 |  | 0.0332 |  | 0.0385 |
|  |  | 252 |  | 0 |  | *0.0460* |  | 0.0026 |  | 0.0095 |  | 0.0128 |
|  |  | 255 |  | 0.9900 |  | 0.8757 |  | 0.9499 |  | 0.9408 |  | 0.9231 |
|  |  | 258 |  | 0 |  | *0.0108* |  | 0.0013 |  | 0.0166 |  | 0.0256 |
|  |  | 261 |  | 0 |  | *0.0054* |  | 0 |  | 0 |  | 0 |
|  |  |  |  |  |  |  |  |  |  |  |  |  |
| psyl44 |  | 166 |  | 0.1608 |  | 0.0568 |  | 0.0990 |  | 0.0822 |  | 0.0513 |
|  |  | 169 |  | 0.0503 |  | 0.0297 |  | 0.0283 |  | 0.0352 |  | 0.0641 |
|  |  | 172 |  | 0.7889 |  | 0.8838 |  | 0.8715 |  | 0.8709 |  | 0.8846 |
|  |  | 175 |  | 0 |  | *0.0243* |  | 0.0013 |  | 0 |  | 0 |
|  |  | 178 |  | 0 |  | *0.0054* |  | 0 |  | 0.0117 |  | 0 |
|  |  |  |  |  |  |  |  |  |  |  |  |  |
| PtTX4001 |  | 199 |  | 0 |  | 0 |  | 0.0013 |  | 0 |  | 0 |
|  |  | 201 |  | 0.1515 |  | 0.2609 |  | 0.1224 |  | 0.1143 |  | 0.1667 |
|  |  | 203 |  | 0 |  | *0.0435* |  | 0.0090 |  | 0.0191 |  | 0 |
|  |  | 205 |  | 0.2071 |  | 0.1929 |  | 0.2384 |  | 0.2262 |  | 0.2821 |
|  |  | 207 |  | 0 |  | 0 |  | 0 |  | 0.0048 |  | 0 |
|  |  | 209 |  | 0 |  | 0 |  | 0 |  | 0.0071 |  | 0.0128 |
|  |  | 211 |  | 0 |  | 0 |  | 0 |  | 0.0071 |  | 0.0128 |
|  |  | 213 |  | 0.1641 |  | 0.0734 |  | 0.1791 |  | 0.1738 |  | 0.0641 |
|  |  | 215 |  | 0.1818 |  | 0.1685 |  | 0.2126 |  | 0.2143 |  | 0.2180 |
|  |  | 217 |  | 0.1995 |  | 0.1766 |  | 0.1546 |  | 0.1524 |  | 0.1410 |
|  |  | 219 |  | 0.0808 |  | 0.0516 |  | 0.0747 |  | 0.0714 |  | 0.1026 |
|  |  | 221 |  | 0 |  | *0.0054* |  | 0 |  | 0 |  | 0 |
|  |  | 223 |  | *0.0101* |  | 0 |  | 0 |  | 0.0048 |  | 0 |
|  |  | 225 |  | 0 |  | *0.0136* |  | 0.0039 |  | 0 |  | 0 |
|  |  | 227 |  | 0 |  | *0.0109* |  | 0.0013 |  | 0.0024 |  | 0 |
|  |  | 229 |  | 0.0051 |  | 0.0027 |  | 0.0026 |  | 0.0024 |  | 0 |

Frequencies of alleles specific to the native or exotic adult samples are highlighted in italics.

**Table S4 (continued).** Allelic frequencies for nSSR loci in *Pinus sylvestris*.

| nSSR | | |  | Sample | | | | | | | | |
| --- | --- | --- | --- | --- | --- | --- | --- | --- | --- | --- | --- | --- |
| Locus |  | Allele |  | Adults | | |  | Seeds |  | Recruits | | |
|  |  |  |  | Native |  | Exotic |  |  |  | R1 |  | R2 |
| PtTX4011 |  | 256 |  | 0 |  | *0.0342* |  | 0.0014 |  | 0.0075 |  | 0.0263 |
|  |  | 258 |  | 0.5969 |  | 0.5124 |  | 0.6554 |  | 0.6508 |  | 0.7105 |
|  |  | 260 |  | 0 |  | *0.0342* |  | 0 |  | 0.0050 |  | 0 |
|  |  | 262 |  | 0.0183 |  | 0.0155 |  | 0.0243 |  | 0.0126 |  | 0 |
|  |  | 264 |  | 0.3848 |  | 0.3665 |  | 0.3176 |  | 0.3216 |  | 0.2632 |
|  |  | 266 |  | 0 |  | *0.0093* |  | 0 |  | 0 |  | 0 |
|  |  | 278 |  | 0 |  | *0.0186* |  | 0 |  | 0.0025 |  | 0 |
|  |  | 280 |  | 0 |  | *0.0093* |  | 0.0014 |  | 0 |  | 0 |
|  |  |  |  |  |  |  |  |  |  |  |  |  |
| SPAC 11.4 |  | 127 |  | 0 |  | 0 |  | 0 |  | 0.0048 |  | 0 |
|  |  | 129 |  | 0.0227 |  | 0.0055 |  | 0.0142 |  | 0.0238 |  | 0.0641 |
|  |  | 133 |  | 0 |  | *0.0027* |  | 0 |  | 0 |  | 0 |
|  |  | 135 |  | 0 |  | *0.0137* |  | 0.0013 |  | 0 |  | 0 |
|  |  | 137 |  | 0.0606 |  | 0.1585 |  | 0.0452 |  | 0.0643 |  | 0.1154 |
|  |  | 139 |  | 0.2121 |  | 0.1093 |  | 0.1576 |  | 0.1929 |  | 0.1667 |
|  |  | 141 |  | 0.0404 |  | 0.1284 |  | 0.0594 |  | 0.0643 |  | 0.0897 |
|  |  | 143 |  | 0.0884 |  | 0.0328 |  | 0.0685 |  | 0.0643 |  | 0.0513 |
|  |  | 145 |  | 0.0101 |  | 0.0574 |  | 0.0284 |  | 0.0191 |  | 0.0385 |
|  |  | 147 |  | 0.0177 |  | 0.0410 |  | 0.0142 |  | 0.0167 |  | 0.0128 |
|  |  | 151 |  | 0.1742 |  | 0.1230 |  | 0.2003 |  | 0.2024 |  | 0.1795 |
|  |  | 153 |  | 0.0202 |  | 0.0383 |  | 0.0258 |  | 0.0191 |  | 0.0256 |
|  |  | 155 |  | 0.0177 |  | 0.0219 |  | 0.0194 |  | 0.0191 |  | 0.0128 |
|  |  | 157 |  | 0.1187 |  | 0.0383 |  | 0.1305 |  | 0.1000 |  | 0.0897 |
|  |  | 159 |  | 0.0354 |  | 0.1175 |  | 0.0246 |  | 0.0262 |  | 0.0256 |
|  |  | 161 |  | 0.1616 |  | 0.0601 |  | 0.1873 |  | 0.1714 |  | 0.1282 |
|  |  | 163 |  | 0.0177 |  | 0.0328 |  | 0.0155 |  | 0.0119 |  | 0 |
|  |  | 165 |  | 0 |  | *0.0191* |  | 0.0013 |  | 0 |  | 0 |
|  |  | 167 |  | 0 |  | 0 |  | 0.0039 |  | 0 |  | 0 |
|  |  | 169 |  | *0.0025* |  | 0 |  | 0.0026 |  | 0 |  | 0 |

Frequencies of alleles specific to the native or exotic adult samples are highlighted in italics.

**Table S5.** Statistical significance, *P-*values and Holm-Bonferroni corrected *P*-values (Holm 1979), of tests of the null hypothesis of equal exotic gene flow rates across *Pinus pinaster* sequential offspring samples. Separate tests were conducted for each sample pair and each exotic gene flow component: male gametic ($\hat{m}_{m}$), zygotic ($\hat{m}_{z}$) and female gametic ($\hat{m}_{f}$). Seeds versus seedling (“R1”, “R2”, “R3”) differences were tested through likelihood-ratio tests, while seedling versus seedling differences were tested through randomization tests (see Material and Methods for details).

|  |  | $\hat{m}_{m}$ | |  | $\hat{m}_{z}$ | |  | $\hat{m}_{f}$ | |
| --- | --- | --- | --- | --- | --- | --- | --- | --- | --- |
| Comparison |  | *P* | *P* (corrected) |  | *P* | *P* (corrected) |  | *P* | *P* (corrected) |
| Seeds vs R1 |  | 0.039 | 0.234 |  | - | - |  | - | - |
| Seeds vs R2 |  | 0.042 | 0.252 |  | - | - |  | - | - |
| Seeds vs R3 |  | 0.263 | 1.000 |  | - | - |  | - | - |
| R1 vs R2 |  | 0.997 | 1.000 |  | 1.000 | 1.000 |  | 0.497 | 1.000 |
| R1 vs R3 |  | 0.538 | 1.000 |  | 0.980 | 1.000 |  | 0.587 | 1.000 |
| R2 vs R3 |  | 0.461 | 1.000 |  | 0.747 | 1.000 |  | 0.581 | 1.000 |

**Table S6.** Statistical significance, *P-*values and Holm-Bonferroni corrected *P*-values (Holm 1979), of tests of the null hypothesis of equal exotic gene flow rates across *Pinus sylvestris* sequential offspring samples. Separate tests were conducted for each sample pair and each exotic gene flow component: male gametic ($\hat{m}_{m}$), zygotic ($\hat{m}_{z}$) and female gametic ($\hat{m}_{f}$). Seeds versus seedling (“R1”, “R2”) differences were tested through likelihood-ratio tests, while seedling versus seedling differences were tested through randomization tests (see Material and Methods for details).

|  |  | $\hat{m}_{m}$ | |  | $\hat{m}_{z}$ | |  | $\hat{m}_{f}$ | |
| --- | --- | --- | --- | --- | --- | --- | --- | --- | --- |
| Comparison |  | *P* | *P* (corrected) |  | *P* | *P* (corrected) |  | *P* | *P* (corrected) |
| Seeds vs R1 |  | 0.125 | 0.375 |  | - | - |  | - | - |
| Seeds vs R2 |  | 0.359 | 1.000 |  | - | - |  | - | - |
| R1 vs R2 |  | 0.526 | 1.000 |  | 0.455 | 0.455 |  | 0.795 | 0.795 |

**Literature cited**

Holm, S. 1979. A simple sequentially rejective multiple test procedure. Scandinavian Journal of Statistics **6**:65–70.

Rannala, B., and J. L. Mountain 1997. Detecting immigration by using multilocus genotypes. Proceedings of the National Academy of Sciences, USA **94**:9197–9201.

Unger, G. M., Vendramin, G. G., and J. J. Robledo-Arnuncio 2014. Estimating exotic gene flow into native pine stands: zygotic vs. gametic components. Molecular Ecology **23**:5435–5447.
